# A Fundamental Investigation of Gas/Solid Heat and Mass Transfer in Structured Catalysts Based on Periodic Open Cellular Structures (POCS)

**DOI:** 10.1021/acs.iecr.1c00215

**Published:** 2021-06-15

**Authors:** Claudio Ferroni, Mauro Bracconi, Matteo Ambrosetti, Matteo Maestri, Gianpiero Groppi, Enrico Tronconi

**Affiliations:** † Laboratory of Catalysis and Catalytic Processes, Dipartimento di Energia, Politecnico di Milano via La Masa 34, 20156 Milano, Italy

## Abstract

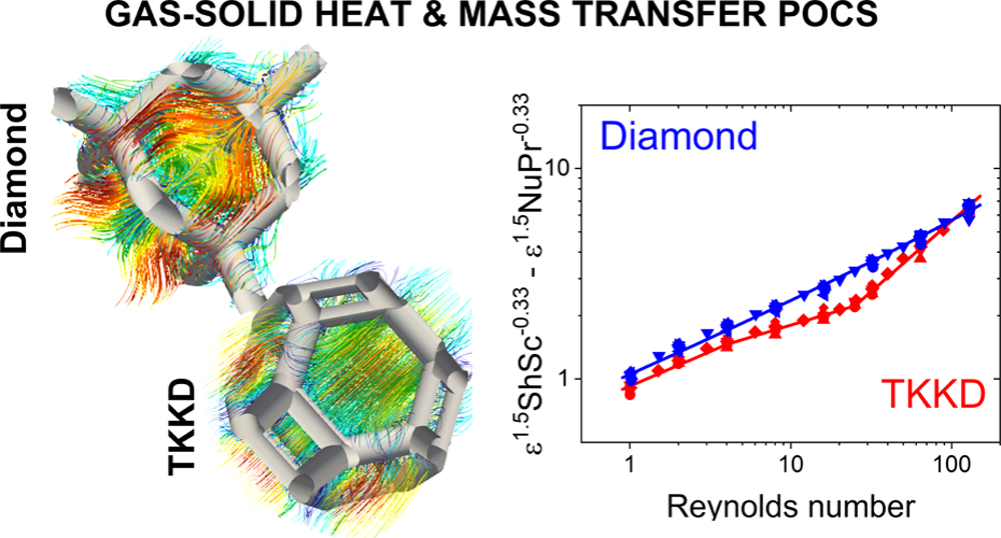

In this work, we investigate the gas–solid heat and mass transfer in catalytically activated periodic open cellular structures, which are considered a promising solution for intensification of catalytic processes limited by external transport, aiming at the derivation of suitable correlations. Computational fluid dynamics is employed to investigate the Tetrakaidekahedral and Diamond lattice structures. The influence of the morphological features and flow conditions on the external transport properties is assessed. The strut diameter is an adequate characteristic length for the formulation of heat and mass transfer correlations; accordingly, a power-law dependence of the Sherwood number to the Reynolds number between 0.33 and 0.67 was found according to the flow regimes in the range 1–128 of the Reynolds number. An additional −1.5-order dependence on the porosity is found. The formulated correlations are in good agreement with the simulation results and allow for the accurate evaluation of the external transfer coefficients for POCS.

## 1 Introduction

Heterogeneous catalysis plays a fundamental role in the context of process intensification, as new catalytic technologies allow to develop more compact, safe, energy-efficient and environmental-friendly processes. Structured catalysts are considered at the heart of this topic, and have been widely employed for several applications affected by heat and mass transfer limitations.^1−4^ Structured catalysts were first introduced in the form of the honeycomb monolith.^5^ The high porosities of such substrates and the typical laminar flow conditions prevailing in the channels enable substantial reduction of pressure drop with respect to conventional packed beds of catalyst pellets despite very high surface area. Because of these features, honeycomb monoliths are the standard catalyst shape in most of the applications related to environmental catalysis.^2^ The growing need of high-performing supports with enhanced volumetric transfer rates led, throughout the past decades, to the research of new and more effective solutions. This problem has been originally tackled by considering open cell foams as a potential innovative catalytic support.^6−10^ Foams are constituted by an ensemble of irregular open cells, reciprocally communicating through pores, and delimited by solid ligaments with variable shape. Foams exhibit high fluid–solid interphase transfer rates, mainly due to the intense mixing provided by their complex solid matrix; however, for the same reason, they exhibit higher pressure drop than honeycombs, thus hindering their direct application at least to environmental catalysis processes.^11^ Recently, Schwieger, Freund, and co-workers^12,13^ introduced the concept of periodic open cellular structures (POCS). They consist of an ordered assembly of interconnected regular unit cells constituted by solid ligaments, or struts, and open windows. The cells have well-defined geometry and fill the space in a regular periodic pattern constituting a three-dimensional frame. The lattice-based structures are characterized by high porosities and thus provide relatively low pressure drop when compared with packed beds.^14,15^ Despite the high porosities, they provide large active surface areas, associated with a complex solid matrix which promotes the fluid mixing. POCS can be manufactured by advanced 3D printing techniques, which offer the opportunity to optimize the support geometry to tailor the design to the specific application and produce exact replicas of the optimized shapes.^13,16−20^ In this view, several unit cell shapes are available in the literature and already reproducible by 3D printing.^21^ A few of these have been considered for chemical reaction engineering applications, namely, the Cubic, the Diamond, the Octet, and the Tetrakaidekahedral (TKKD) unit cells.^12−14,16−19,22,23^ Klumpp et al.^16^ proposed a fully theoretical derivation of the ideal Cubic unit cell geometrical features, providing the expressions for the evaluation of the porosity, the specific surface area, and the window length as a function of the cell tilting. Horneber^24^ additionally provided the geometrical models of the Diamond and the TKKD ideal unit cells. The cells were described by merging cylindrical struts with spheres for the strut junctions in the nodes.

POCS may find several potential applications in the intensification of chemical processes. Because of their discussed geometrical features, POCS can potentially provide intense high gas–solid transfer rates, and thus, they may find successful application for processes under external mass transfer control (e.g., highly exothermic partial oxidation of hydrocarbons).^25,26^ At the same time, for the same reason, catalytically activated ceramic POCS^19,20^ look attractive for exhaust aftertreatment systems.^27,28^ Thanks to their continuous, fully interconnected solid matrix, metallic POCS can offer high effective thermal conductivity and look attractive for industrial processes and especially in chemical syntheses, where the heat management is key.^13,17,29^

Few studies in the literature report the investigation of the fluid–solid transport properties of POCS. From the experimental standpoint, gas–solid heat and mass transfer in POCS has been investigated for a reduced number of cell shapes and geometries.^23,30^ Balzarotti et al.^23^ investigated external mass transfer in Cubic cell POCS manufactured by investment casting and catalytically activated with Pd/CeO_2_ by spin coating for CO oxidation. They derived a Sh-Re correlation to model gas–solid mass transfer rates in cubic cell POCS as a function of the support morphological parameters and the flow condition. The work highlights the potential of POCS in the intensification of mass transfer limited catalytic applications, as they offer higher transfer rates than the state-of-the-art honeycombs currently available on the market. Rebelo et al.^30^ investigated pressure drop and gas–solid interphase heat transfer in cubic cell POCS, 3D printed in aluminum alloy. The authors modeled the effect of the main considered geometrical design parameters (the strut length, or cell size; the porosity; and the cell orientation) on the pressure drop and the overall heat transfer. The pressure drop was described as a parabolic function of the velocity, which was compared to the Ergun equation for packed beds. Ergun coefficients appeared to overestimate POCS pressure drop, in complete accordance with other authors’ findings.^16,31^ A generalized correlation for the evaluation of the Nusselt number at high Reynolds number was also reported. Chaudhari et al.^32^ experimentally investigated the performance of the Octet-truss lattice (OTL) structure made of AlSiMg alloy, examining pressure drop, and convective heat transfer. From pressure drop measurements on samples at different porosity, the authors obtained the permeability and the inertial coefficient of an Ergun-like correlation. They, thus provided friction factor correlations for their specific OTL samples, whereas a generalized correlation between the pressure drop and the morphological features was not obtained. Similarly, the authors proposed Nu-Re correlations for their specific OTL structures, although the effect of the morphological parameters was not completely discussed.

The evaluation of the transport properties by experiments requires specialized infrastructures, with significant cost, and is characterized by slow procedures which hinder the examination of the multiple solutions available for lattice structures’ design. Moreover, the experimental investigation is constrained by the limits of the current 3D-printing technology, which hinders the evaluation of the effect of single geometrical parameters on the properties of interest in the proximity of the manufacturability range. However, numerical detailed simulations can be considered as synthetic experiments which enable the investigation of the properties of interest in any kind of innovative material in advance of its manufacturing, enabling to examine a broad range of working conditions and to assess the effect of single morphological parameters without any mutual interference.^9,11^ Owing to these benefits, computational fluid dynamics (CFD) has gathered high interest throughout the past decade for the investigation of cellular materials. CFD has proven to be a reliable tool, which has already been exploited by our group for the analysis of microchannels^33^ and open-cell foams.^9^ In facts, CFD grants a complete accordance with experimental measurements, allowing for the deep analysis of both the flow field and the transport mechanisms, which enables the derivation of dimensionless engineering correlations for the transport properties.^9,11^ Regarding POCS, Kuipers and co-workers numerically investigated the dispersion mechanism inside the TKKD unit cell, considering different porosities at various Péclet numbers.^34^ Then, they numerically investigated the influence of the flow rate on the mass transfer in the same cell at fixed porosity, at moderate Reynolds numbers, providing a criterion to determine the controlling regime in the conditions of interest.^35^ Sun et al.^22^ proved that, although the considered TKKD and foam structures with equivalent porosity and cell size exhibited similar pressure drops, the TKKD offered significantly higher interphase heat transfer rates, essentially due to the larger TKKD specific surface area. Papetti et al.^19^ performed a coupled numerical and experimental investigation of mass transfer in open cell lattice structures with several different cell shapes, showing the enhanced transport properties of POCS when compared with honeycomb monoliths. They highlighted that the cell shape plays a major role in determining the mass transfer rates and proved that the TKKD and the tilted Cubic cells offer the highest rates with respect to the simple cubic cell. By further evaluation on the trade-off between the increased mass transfer and pressure drop, they showed that highly porous cellular structures may offer significantly better overall performances than honeycomb supports.

Literature works highlight the POCS potential for the intensification of gas–solid heat and mass transfer limited catalytic applications. Nonetheless, a few pieces of information are still lacking, hindering the proper definition of their performance in real applications. In particular, a systematic investigation of the gas–solid transfer properties, devoted to their full characterization as a function of the fluid conditions and of the support morphological properties, is missing.

This work aims at carrying out a comprehensive analysis of the fluid–solid transport in POCS with TKKD and Diamond unit cells, assessing the influence of the support morphology (i.e., cell size, porosity, cell shape) and flow conditions on the dimensionless transport coefficients.

The investigation of the gas–solid transport is carried out aiming at the development of engineering correlations to allow for the estimation of heat and mass transfer coefficients, which can be exploited to compare different technologies and enable the design of novel chemical reactors based on these technologies. In doing so, and in analogy with our previous work related to foams,^9^ CFD simulations are employed as in silico experiments which provide the data required for the development of CFD-based engineering correlations. Simulations on virtually generated ideal POCS have been performed either by using CO oxidation as a test surface reaction for the evaluation of external mass transfer properties or by prescribing a uniform wall temperature for the evaluation of gas–solid interphase heat transfer. In this regard, periodic boundary conditions have been introduced to model POCS in periodic fully developed flow and heat and mass transfer. Originally proposed by Patankar et al.^36^ for incompressible flows in bidimensional systems, periodic boundary conditions have been extended to model reactive compressible flows in the three-dimensional space, and represent an element of novelty with respect to our previous work for foams.^9^ The effects of the flow conditions and of the geometrical features (porosity, cell size) on the dimensionless transport coefficients have been addressed. We have observed in fact that both the cell shape and the porosity have a major influence on the gas–solid transport properties. As a result, we have derived engineering correlations that are able to represent the effects of geometrical properties and flow conditions on the transport properties. On the basis of this analysis, we have performed a comparison of the volumetric mass transfer rates associated with POCS, with open-cell foams and with square channel honeycombs. Mass transfer rates of cellular structures are shown to strongly outperform industrial honeycombs, and POCS are shown to exhibit even better performances than foams. The derived engineering correlations enable the exploitation of these innovative enhanced catalyst supports, paving the way to the next generation of catalytic reactors.

## 2 Methods

In this section, we provide an overview of the numerical CFD methodology implemented for the investigation of gas-to-solid heat and mass transfer properties of periodic open cellular structures (POCS). The investigation was performed on the ideal Tetrakaidekahedral (TKKD) and the Diamond unit cells. The geometrical models exploited in this work are initially illustrated. The adopted CFD model are then briefly discussed. Finally, we discuss the 1D reactor model applied to the analysis of the results.

### 2.1 Geometrical Models

**Figure 1:**
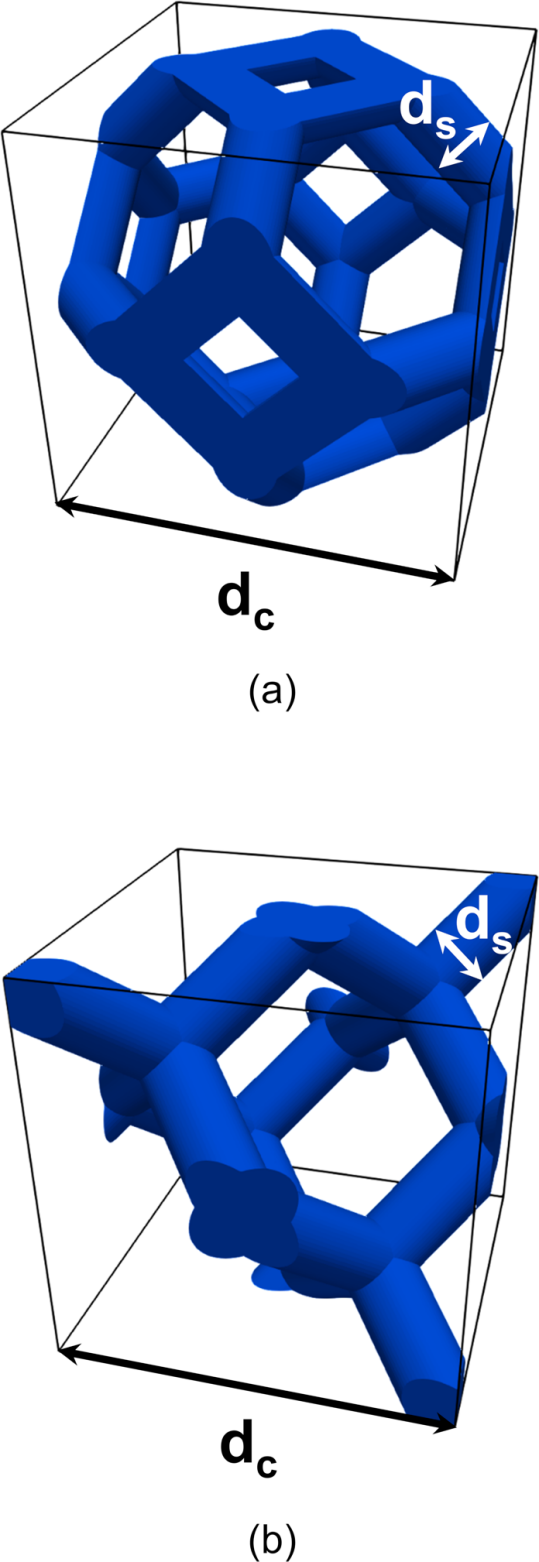
POCS unit cells: (a) Tetrakaidekahedral unit cell, (b) Diamond unit cell. The cell size d_c_ and the strut diameter d_s_ are highlighted in black and white, respectively.

Detailed geometrical models are required to provide a systematic analysis of transport phenomena in porous structures. In this work, the Tetrakaidekahedral (TKKD) and the Diamond unit cells are considered (Figure 1). Generally, it is possible to identify six geometrical parameters in POCS, namely, the strut diameter, the strut length, the cell size, the surface area, the porosity (or its complement, the solid volume fraction), and the mean window diameter, with two of them independent.^24^ In this work, the cell size and the porosity are chosen as the design parameters of the structures. For further analyses, two additional geometrical parameters, the strut diameter (d_s_), and the specific surface area (S_v_) have a great significance. Therefore, their dependency on the cell size and the porosity is briefly discussed.

Horneber^24^ proposed a fully theoretical characterization of TKKD and Diamond POCS geometrical properties, providing the models of these structures with circular struts with constant cross-sectional area.

In this view, the Diamond unit cell geometrical model developed by Horneber^24^ is adopted for the description of the diamond lattice geometrical features. However, the TKKD unit cell model shows significant deviations between the theoretical geometrical properties and the computed values at decreasing porosity, with a maximum error between the predicted porosity and the measured value attaining 5.3% at ε = 0.7 (see Supporting Information, Section 1.1 for additional details). In this view, we have hereby derived a geometrical model for the TKKD based on the model proposed by Ambrosetti et al.^37^ for open-cell foams. The authors proposed a theoretical derivation of the geometrical properties of open-cell foams based on a TKKD unit cell with struts with variable cross-section. In this work and in analogy with ref (38), we adapted the existing model to the ideal TKKD unit cell considering instead circular struts with constant cross-section. More details on the developed geometrical model are reported in the Supporting Information, Section 1.1, along with its validation and the comparison with the model proposed by Horneber.^24^

The equations for the description of the porosity and the specific surface area of the TKKD and the Diamond unit cells are reported in Table 1. Table 1 provide the explicit dependency of the porosity and the specific surface area to the strut diameter and cell size. When considering the cell size and the porosity as design parameters, the strut diameter is implicitly given by the cubic equation, and the specific surface area is evaluated accordingly.

**Table 1:** Equations for the Evaluation of the Geometrical Properties of TKKD and Diamond Unit Cell POCS

	TKKD	diamond^24^
porosity	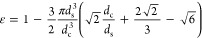	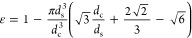
specific surface area	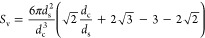	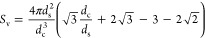

**Figure 2:**
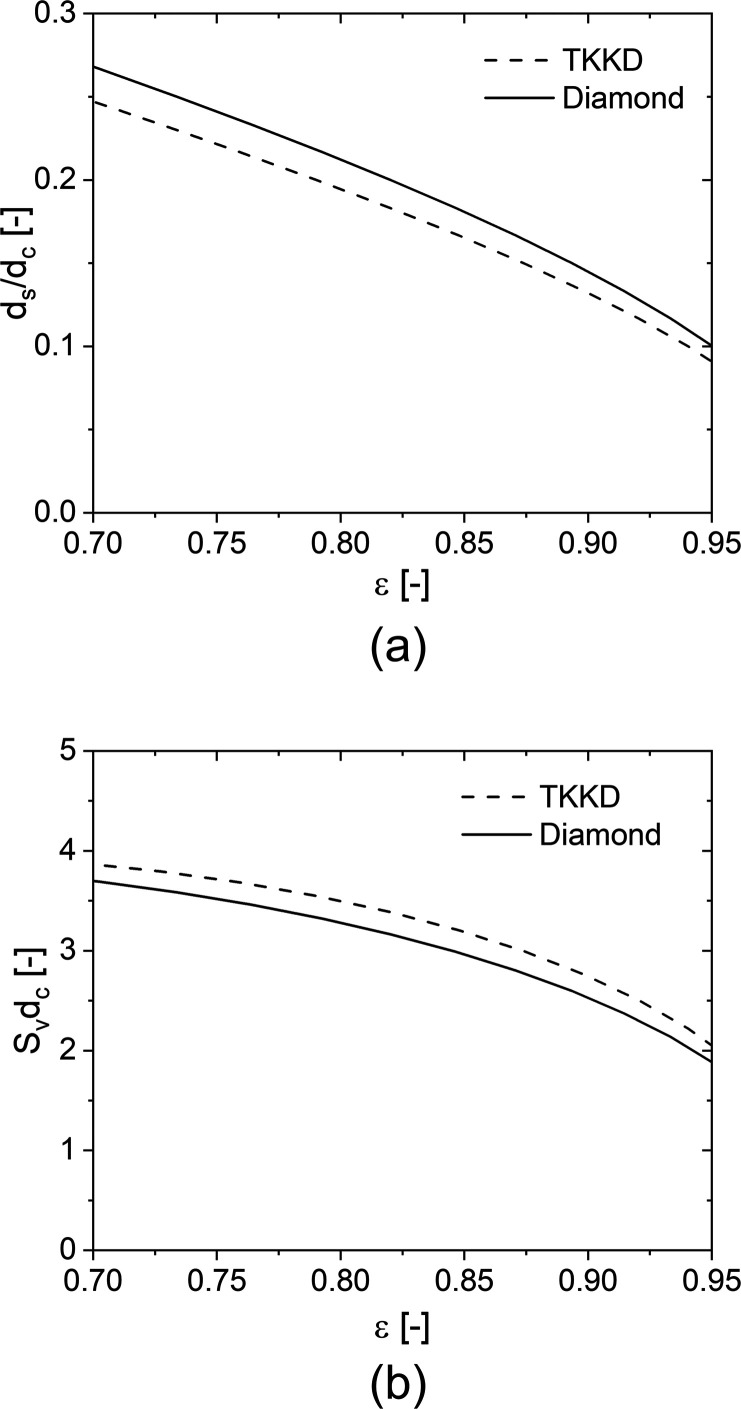
Dimensionless strut diameter against porosity (a) and dimensionless specific surface area (b) for the TKKD and the Diamond unit cell POCS.

The strut diameter and the specific surface area are directly and inversely proportional to the cell size, respectively, as already reported by Tronconi and co-workers^9,37^ in the case of open-cell foams. Therefore, the dimensionless quantities d_s_d_c_^–1^ and S_v_d_c_ can be plotted against the porosity. Figure 2a shows that the strut diameter of both TKKD and Diamond decreases almost linearly for ε < ∼0.85, while a nonlinear dependency is more evident at higher porosity. It is worth noticing that the TKKD unit cell exhibits thinner struts and, consequently, higher specific surface area than the Diamond unit cell at the same porosity, as shown in Figure 2b.

The geometrical features of the virtually generated domains used for the analysis of gas–solid heat and mass transfer are reported in Table 2 and Table 3.

**Table 2:** Geometrical Features of the Virtually Generated Diamond Unit Cell POCS

diamond samples				
	*d*_c_ [mm]	ε [-]	*d*_s_ [mm]	*S*_v_ [m^–1^]
Dia-0.70_3	3.000	0.700	0.804	1233
Dia-0.75_3	3.000	0.750	0.723	1174
Dia-0.80_3	3.000	0.800	0.637	1094
Dia-0.85_3	3.000	0.850	0.543	988
Dia-0.90_3	3.000	0.900	0.435	844
Dia-0.95_3	3.000	0.950	0.301	628
Dia-0.85_1	1.000	0.850	0.181	2965
Dia-0.85_4	4.000	0.850	0.723	741
Dia-0.85_8	8.000	0.850	1.447	371

**Table 3:** Geometrical Features of the Virtually Generated TKKD Unit Cell POCS Samples

TKKD samples				
	*d*_c_ [mm]	ε [-]	*d*_s_ [mm]	*S*_v_ [m^–1^]
TKKD-0.70_3	3.000	0.700	0.742	1289
TKKD-0.75_3	3.000	0.750	0.665	1240
TKKD-0.80_3	3.000	0.800	0.584	1167
TKKD-0.85_3	3.000	0.850	0.496	1063
TKKD-0.90_3	3.000	0.900	0.396	915
TKKD-0.95_3	3.000	0.950	0.273	687
TKKD-0.85_1	1.000	0.850	0.165	3189
TKKD-0.85_4	4.000	0.850	0.661	797
TKKD-0.85_8	8.000	0.850	1.322	399

### 2.2 CFD Modeling

The investigation of the gas–solid mass transfer in POCS is performed using the catalyticFOAM^39^ framework, a numerical tool which couples the solution of the Navier–Stokes equation and the species mass balances with the detailed characterization of the surface chemistry. The exhaustive description of the mathematical models and the numerical methods exploited for the solution is shown by Maestri et al.^39^ The proposed methodology has been already employed and validated in the context of open-cell foams.^9^ In particular, at the catalytic surface, the net production or consumption rates are equal to the local mass fluxes due to species diffusion. To achieve an external mass transfer fully limited regime, an infinitely fast heterogeneous reaction is imposed at the catalytic surface, to reach a null surface concentration of the mass transfer limited reactant.

A second-order upwind scheme (linear upwind) was adopted for the discretization of the convective terms, while a pure second-order scheme was employed for the diffusive terms. Operating conditions imposed in the performed CFD simulations are summarized in Table 4.

**Table 4:** Simulation Conditions Set in the CFD Mass Transfer Simulations

mass transfer simulations			
feed temperature		293.15	[K]
outlet pressure		1.0	[bar]
feed mass fraction	CO	0.0292	[-]
	O_2_	0.2262	[-]
	N_2_	0.7446	[-]

The investigation of gas-to-solid heat transfer in POCS is performed as well using the catalyticFOAM framework. Gas–solid heat transfer is evaluated by imposing a constant and uniform wall temperature at the solid wall, enabling the evaluation of the Nu_T_.^40^ A second-order upwind scheme was adopted for the discretization of the convective terms, whereas a pure second-order scheme was adopted for the diffusive terms. Operating conditions imposed in the performed CFD simulations are summarized in Table 5.

**Table 5:** Simulation Conditions Set in the CFD Heat Transfer Simulations

heat transfer simulations		
feed temperature	291.15	[K]
outlet pressure	1.0	[bar]
wall temperature	293.15	[K]

The thermodynamic and transport properties adopted in this work for the analysis of the CFD and experimental data and for the CFD simulations are evaluated by means of the catalyticSMOKE^39^ and OpenSMOKE++ libraries.^41^ In particular, the thermodynamic properties of the gas species, estimated following the approach proposed by Gordon and McBride,^42^ are exploited to calculate the gas mixture properties using the Gibbs theorem. The transport properties are evaluated according to standard kinetic theory expressions.^43,44^

POCS fluid dynamic behavior is investigated in the fully developed regime, neglecting any influences due to entrance effects and lateral constraints. To describe and analyze the fully developed regime in POCS, periodic boundary conditions are implemented for all the relevant variables.^36,45,46^ Under fully developed flow conditions, the physical properties of interest (i.e., fluid specific flow rate, pressure, composition, and temperature) are reduced to periodic functions in the streamwise and transverse directions. Details on the implementation of periodic boundary conditions for compressible flows in the three-dimensional space are reported in the Supporting Information, Section 1.3. At the solid wall boundary, a no-slip condition and a zero gradient condition are imposed for the fluid velocity and the pressure, respectively. As discussed above, a virtually infinitely fast reaction is imposed at the solid wall boundary to reach a null concentration of the mass transfer limited reactant in mass transfer simulations, whereas a constant uniform temperature is prescribed at the solid wall in heat transfer simulations. Details on the computational methods, including the domain and grid generation, and the mesh independence analysis, are available in the Supporting Information, Section 1.2 and Section 1.4.

Steady and unsteady CFD simulations are performed to model pure laminar and unsteady laminar regimes, respectively. In the second case, the fundamental quantitative information provided by transient simulations is time-averaged after reaching steady-state, which is typically achieved in a simulation time equal to 3 residence times. The time-averaging procedure is performed on a time span equal to 3 residence times as well, consecutive to the prior 3.

### 2.3 1D Reactor Model

In this work, a conventional 1D heterogeneous steady-state isothermal plug flow reactor model is employed for the interpretation of the simulation results.^5,47,48^ As discussed in Section 2.2, when considering the mass transfer analysis, CFD simulations are carried out in isothermal conditions. For the interpretation of the results, the energy balance is thus not considered. In CFD simulations, external mass transfer control is reached by imposing a virtually infinitely fast reaction at the catalytic surface. Consequently, the mass transfer limited reactant (CO for our test conditions) approaches a null surface concentration. Therefore, to evaluate the mass transfer coefficient, only the fluid phase mass balance for the mass transfer limited reactant is necessary. The steady-state mass balance for the limiting reactant in full external mass transfer control can be expressed as follows:
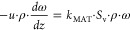
1where *u* is the superficial velocity, ρ is the working fluid density, ω is the mass fraction of the mass transfer limited reactant, *z* is the streamwise coordinate, *k*_MAT_ is the mass transfer coefficient, *S*_v_ the support specific surface area. Equation 1 is developed under the assumption of constant transport properties and uniform flow distribution, which are reproduced by performing the numerical simulations in diluted conditions. POCS specific surface area is given by the models expressed in Section 2.1. Introducing the Sherwood number, eq 1 can be reformulated as follows:
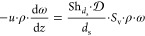
2where Sh_*d*_s__ is the Sherwood number based on the strut diameter *d*_s_ as characteristic length and  is the reactant diffusivity. Integrating the previous equation over the reactor length (*z* = *L*), and solving for the Sherwood number, the following expression is obtained:
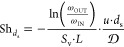
3where the subscripts IN and OUT define the reactor inlet and outlet, respectively. On introducing the definition of conversion X:
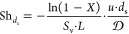
4

The quantities ω_IN_ and ω_OUT_ are computed as cup-mix averages at the inlet and outlet section of the domain:^40^
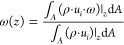
5

In complete analogy to the gas-to-solid mass transfer, the convective heat transfer can be interpreted by the conventional 1D heterogeneous steady-state isothermal plug flow reactor model as well. In the heat transfer analysis, POCS are immersed in a homogeneous inert stream at a different temperature with respect to the solid surface temperature. A small temperature difference (2 K) was imposed between the working fluid at the inlet section of the domain and the solid wall surface to achieve constant fluid physical properties. The energy balance for the solid phase is redundant when considering an imposed uniform temperature profile on the solid. Therefore, for the interpretation of the simulation results, only the energy balance on the fluid phase is considered, in analogy to the methodology adopted in the mass transfer analysis. In this view, eq 3 reads as follows:
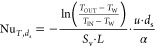
6where Nu_*d*_s__ is the constant-wall-temperature Nusselt number having the strut diameter *d*_s_ as characteristic length, α is the thermal diffusivity, *T*_W_ is the wall temperature and the quantities *T*_IN_ and *T*_OUT_ are temperatures evaluated at the inlet and at the outlet sections of the domain, respectively, and they are computed as cupmix averages:
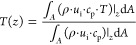
7

Equations 4 and 6 allow us to evaluate the average dimensionless transport coefficient, lumping the local variations due to the support morphology into a single parameter. Owing to periodicity conditions, the mean dimensionless transport coefficient in POCS corresponds to the average value computed across an integer number of unit cells in fully developed flow conditions and either fully developed concentration profile or fully developed temperature profile respectively in mass transfer cases and heat transfer cases (asymptotic conditions).

## 3 Numerical Results

In this section, we show the results of the numerical simulations performed to evaluate the external transport properties of POCS. The geometrical features of the virtually generated domains used for the analysis are reported in Table 2 and Table 3. Samples have a constant circular strut cross-section, and the influence of the strut shape on the properties of interest is not examined. A preliminary analysis has addressed the determination of the Representative Elementary Volume (REV) to provide the proper description of the transport phenomena inside POCS. Then, we examine the effects of the flow rate and of the relevant geometrical parameters on the dimensionless mass transfer coefficient. Simulations are performed considering gas phase with transport properties in the range of 0.7 ≤ Sc (or Pr) ≤ 1.5. Finally, we discuss the analogy between the gas-to-solid mass and heat transfer in POCS.

### 3.1 Appraisal of the Representative Elementary Volume

The determination of the representative elementary volume (REV) is required to properly describe the POCS behavior. As the POCS under investigation are constituted by a 3D arrangement of unit cells with size *d*_c_, the assessment of the REV is performed by estimating the required number of unit cells along the axial and transverse coordinates to obtain dimensionless transport parameters that are independent from the size of the control volume considered. In this view, several simulations have been performed on samples with different size, changing the number of POCS cells in the stream-wise and transversal directions. Periodic fully developed flow conditions have been achieved by imposing the periodicity boundary conditions mentioned in Section 2.2.

In contrast to open-cell foams, which require a REV consisting of several cells to obtain a reliable estimation of the geometry,^9^ the POCS unit cell represents the minimum REV from a geometrical standpoint and thus it has been considered as a starting point in this analysis. Hence, the REV assessment has been performed by starting from a minimum REV of 1 unit cell and by progressively increasing the domain up to 3 cells in the streamwise direction and 2 cells in the transverse direction. The mass transfer simulations are carried out at the conditions listed in Table 4.

**Figure 3:**
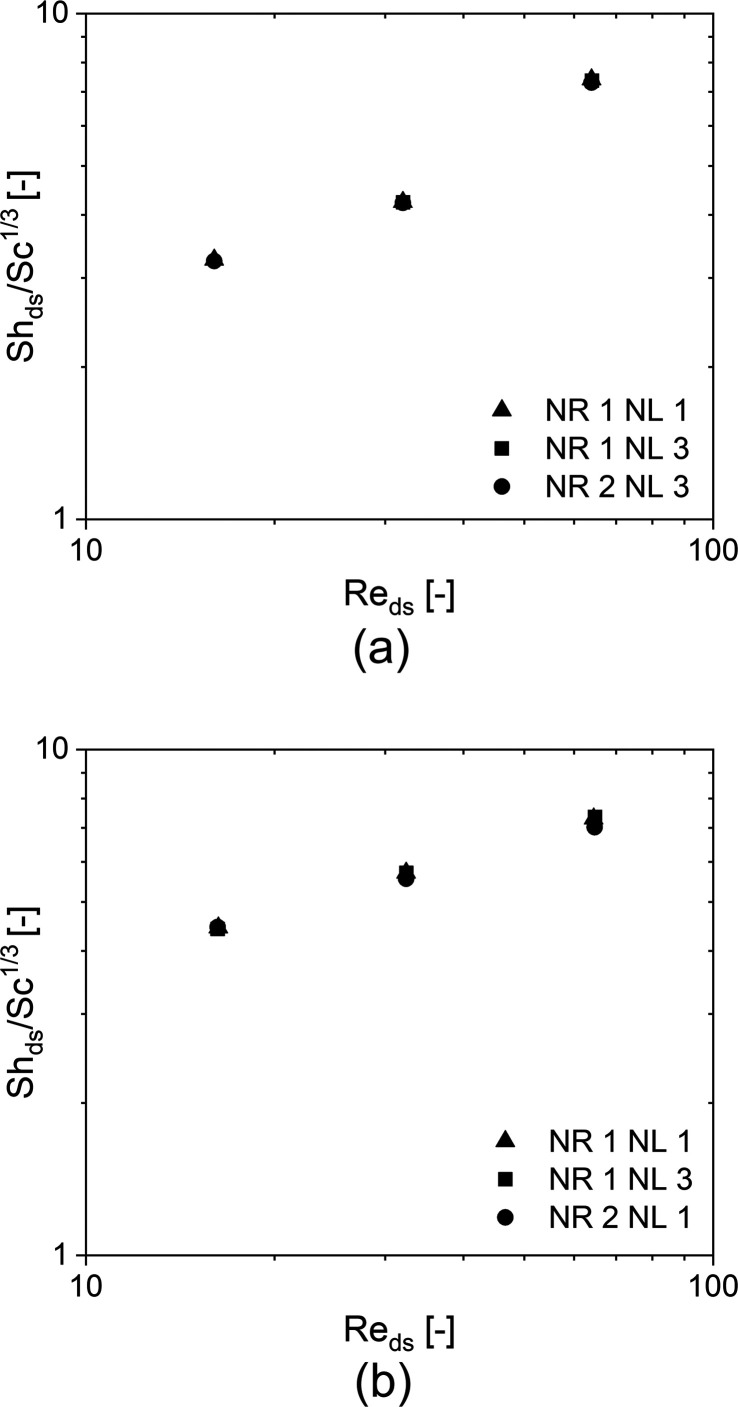
Effect of the sample size on the Sherwood number upon changing the number of cells along the streamwise (NL) and transverse (NR) directions for (a) a TKKD unit cell POCS and (b) a Diamond unit cell POCS. *d*_c_ = 3 mm and ε = 0.7.

Figure 3 reports the Sherwood number against the Reynolds number for the (a) TKKD and the (b) Diamond samples with increasing size. The analysis shows that the POCS unit cell is representative of the entire lattice both from a geometrical and from a fluid dynamic standpoint. Moreover, this condition holds regardless of the flow condition, that is, both in pure laminar and in unsteady laminar conditions.

### 3.2 Effect of the Flow Rate

**Figure 4:**
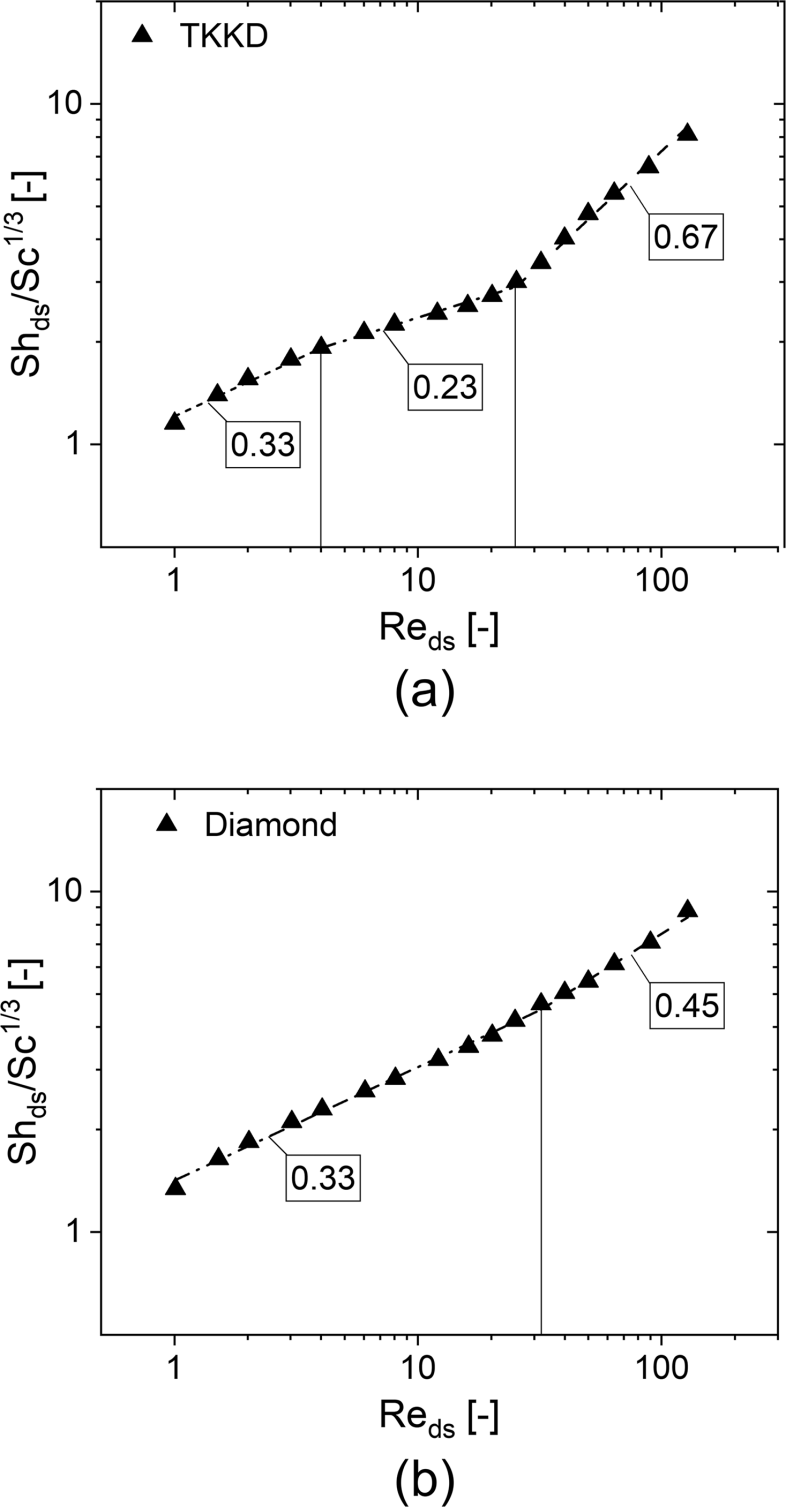
Sherwood numbers against the Reynolds numbers for (a) the TKKD unit cell POCS and (b) the Diamond unit cell POCS. The samples are characterized by cell size *d*_c_ = 3 mm, circular struts with constant cross-section and porosity ε = 0.850.

In this section, the effect of the flow rate on the external mass transfer properties is discussed. The analysis has been performed on samples with 6 porosities, ranging from 0.7 to 0.95, and 4 cell sizes, ranging from 1 mm to 8 mm, for both the TKKD and the Diamond unit cells. The geometrical properties of the POCS samples used in the current analysis are summarized in Table 2 and Table 3. The effect of the flow rate is discussed for samples with porosity ε = 0.85, cell size *d*_c_ = 3 mm and constant circular strut section for both the TKKD and the Diamond unit cell POCS. Analogous behavior has been observed for the other geometries. Figure 4 shows the Sherwood number against the Reynolds number evaluated for (a) the TKKD and (b) the Diamond cell. The analysis reveals that the Sherwood number increases, as expected, with growing flow rate for both the geometries. However, different behaviors can be observed according to the unit cell considered.

**Figure 5:**
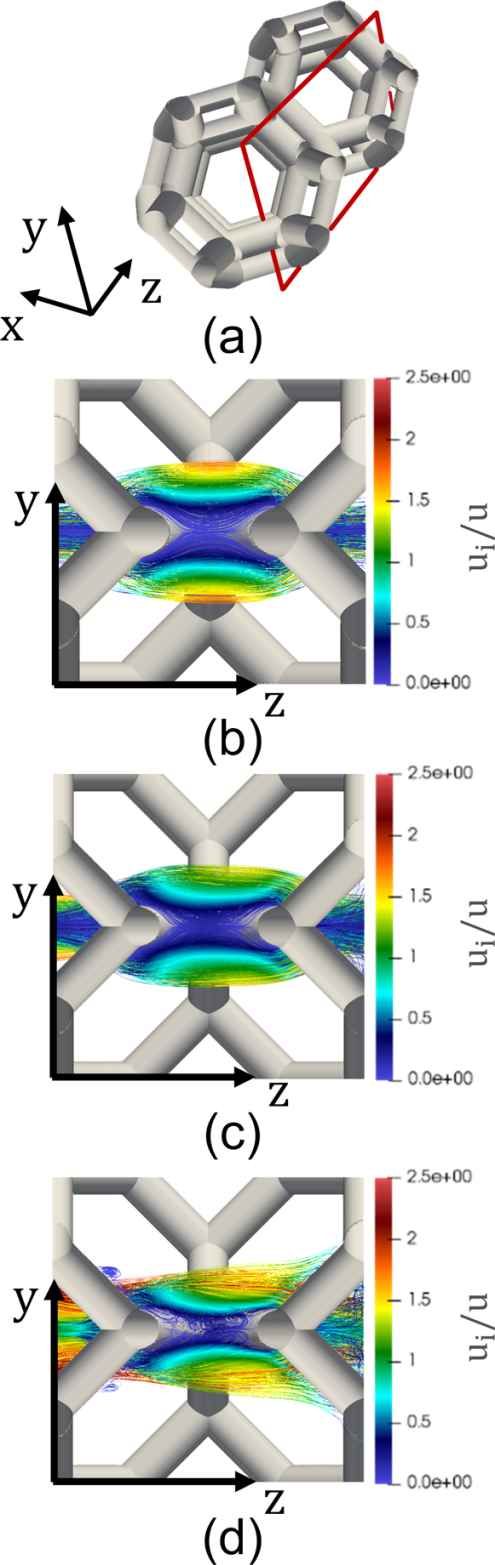
(a) Solid surface along with the projected plane (highlighted in red). Streamlines of the dimensionless fluid velocity field *u*_i_ normalized by the superficial velocity *u* crossing a pair of tilted struts of the TKKD unit cell at (b) Re_ds_ = 1, (c) Re_ds_ = 8, and (d) Re_ds_ = 32. The streamlines are colored as a function of the dimensionless fluid velocity.

The TKKD shows three different dependencies of the Sherwood number on the Reynolds number, as highlighted by the power-law fits (i.e., lines) in Figure 4a corresponding to the following Re intervals: first interval 1 ≤ Re_ds_ ≤ 4, second interval 4 ≤ Re_ds_ ≤ 25, third interval 25 ≤ Re_ds_ ≤ 128. For 1 ≤ Re_ds_ ≤ 4, a creeping viscous flow regime establishes inside the TKKD, as shown in Figure 5b*.* The streamlines of the flow field are adherent to the solid surface and axisymmetric with respect to the direction of the flow. Such behavior is in analogy with flow condition around cylinders^49^ and in tube banks^50^ in the discussed Re range, for which the authors propose a dependency of the Sherwood number to the Reynolds number raised to the power of 1/3. In this work, the best fit of the data set leads to a dependency of the Sherwood number on the Reynolds number raised to the power of 0.35, close to the theoretical value 1/3, which is hereby adopted for the TKKD unit cell in the range of 1 ≤ Re_ds_ ≤ 4. Such dependency arises for foams as well in the same flow regime, as reported in our previous work.^9^ At intermediate Reynolds numbers (i.e., 4 ≤ Re_ds_ ≤ 25), a second regime is established, characterized by a weaker sensitivity of the mass transfer on increasing Re_ds_. This effect may be ascribed to the mutual masking/hiding of the struts lined up along the streamwise direction, as shown in Figure 5c. The fluid flow is confined by the open hexagonal and square windows, whereas in the regions delimited by struts lined up in the flow direction, the fluid stagnates (see blue region in Figure 5c), causing a local reduction of the convective phenomena and thus the mass transfer to the reactive surface. In this view, the entire range of 1 ≤ Re_ds_ ≤ 25 can be considered as a single laminar regime; however, for 4 ≤ Re_ds_ ≤ 25, the mutual hiding of lined up struts moderates the mass transfer increase with the Re_ds_ increase. This phenomenon can be explicated with the decaying of the theoretical exponent 1/3 to the 0.23 obtained by power law fit in the range of 4 ≤ Re_ds_ ≤ 25 and reported in Figure 4a. This hypothesis is supported by the small deviations of the exponent 0.23 obtained by fitting the mass transfer data in the range 4 ≤ Re_ds_ ≤ 25 at different porosity: at ε = 0.7, the fitting of the Sherwood number data set provides a dependency of the Reynolds number raised to the power of 0.22, whereas at ε = 0.95, a slightly higher exponent equal to 0.25 is found. At lower porosity, the struts thicken, and the regions of fluids delimited by struts lined up in the flow direction (see blue region in Figure 5c) in turn thicken. However, by increasing the porosity the struts tighten and the stagnation zones in turn tighten. This interference is reported for periodic arrays of aligned cylinders in cross-flow as well, both in line and staggered configurations in pure laminar regime.^50,51^ Indeed, for an infinite row of cylinders, which is mimicked for instance by means of periodic boundary conditions by Ishimi et al.,^50^ the Sherwood number mildly increases with the increase of Re, as cylinders hide one another in wakes along the streamwise direction.

By further increasing the mass flow rate (i.e., Re_ds_ > 25), a steeper increase of the mass transfer rate is observed. In this regime, a jet-like flow crosses the central square window and the hexagonal windows constituting the TKKD frame, as shown by Das et al.^52^ Consequently, the stagnation zones placed between consecutive struts tighten because of the increase of the local velocity close to the struts (Figure 5d), and the partially hidden struts become more exposed to the fluid flow. At Re_ds_ > 25, the Sherwood number fit leads to a 0.67-order dependency on Re_ds_. Such dependency is representative of the unsteady laminar regime, hereby analyzed in the range 25 ≤ Re_ds_ ≤ 128. The steep increase of the mass transfer rate with the increase of the flow rate in the unsteady laminar regime, represented by the exponent 0.67, compensates the weak dependency 0.23 observed in the pure laminar regime, which may be caused by the disappearance of the effect of struts masking/hiding (Figure 5d). The unsteady laminar regime precedes the fully turbulent flow condition appearing at higher Re.^53,54^ In this view, Pedras and Lemos^53^ recognized the post-Forcheimer flow regime (unsteady laminar flow) in porous media in the range of 150 < Re_p_ < 300, with Re_p_ being the Reynolds number referring to the pore size, corresponding here to ∼30 < Re_ds_ < ∼ 60. Along the same lines, the transition between pure laminar regime and unsteady laminar regime for a single cylinder in cross-flow is reported at Re_D_ = 40, with Re_D_ being the Reynolds number referred to the diameter of the cylinder. The critical Reynolds number of the single cylinder in cross-flow is thus comparable in magnitude to the transitional Reynolds number hereby reported for the TKKD unit cell.

**Figure 6:**
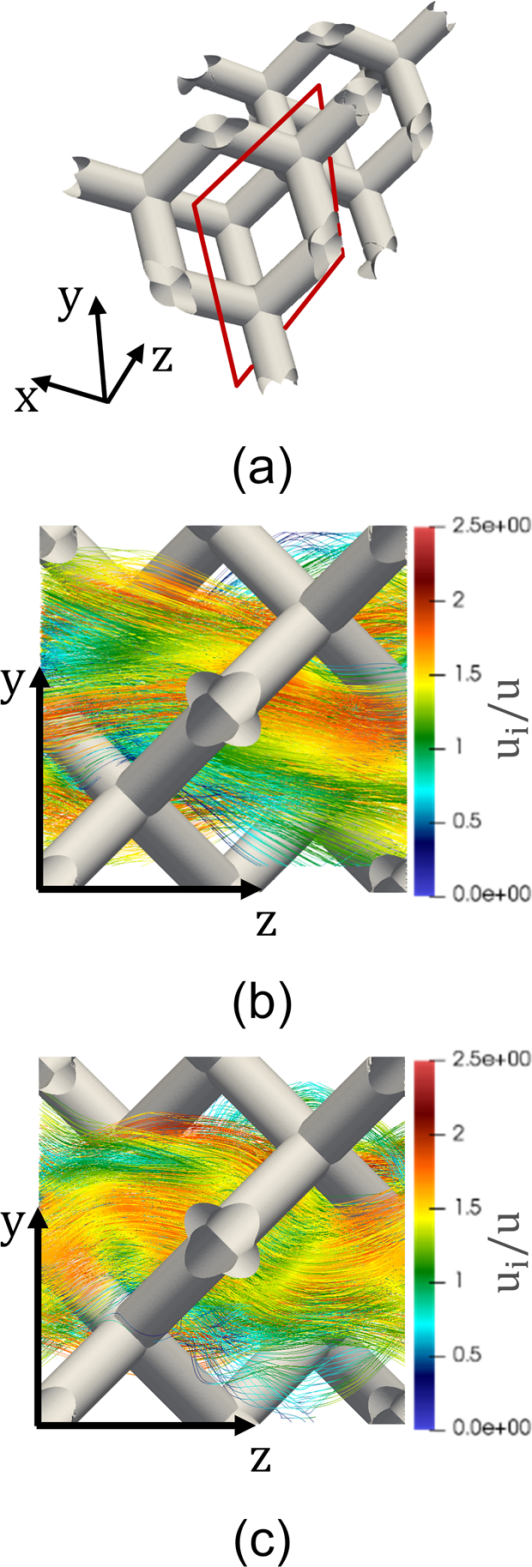
(a) Solid surface along with the projected plane (highlighted in red). Streamlines of the dimensionless fluid velocity field *u*_i_ normalized by the superficial velocity *u* inside the Diamond unit cell at (b) Re_ds_ = 16 and (c) Re_ds_ = 64. The streamlines are colored as a function of the fluid velocity.

The Diamond unit cell (Figure 4b exhibits two different regimes, namely, a full laminar regime at low flow rate (Re_ds_ ≤ 32) and an unsteady laminar regime at Re_ds_ > 32, according to our fluid dynamic simulations. For 1 ≤ Re_ds_ ≤ 32, a laminar flow regime establishes inside the Diamond unit cell, and such behavior is once again comparable to the behavior of cylinders^49^ in cross-flow and in tube banks^50^ in the discussed Re range, for which a dependency of the Sherwood number to the Reynolds number raised to the power of 1/3 was found. Such dependency arises for foams as well in the same flow regime.^9^ Differently to the TKKD, no detrimental effect due to struts reciprocal masking can be observed on the convective mechanism in the Diamond unit cell in the pure laminar regime, and such difference may be ascribed to the great difference between the two cells morphology. The Diamond struts aligned in the flow direction are separated by a greater distance than in the TKKD, as this distance is comparable to the cell size. Contrarily to the TKKD unit cell, the Diamond unit cell is made of nonplanar, hexagonal windows tilted with respect to the flow direction (see Figure 6a). These openings offer low resistance to the flow direction when compared to the TKKD square windows (see, Figure 5a). As a result, the fluid dynamics inside the Diamond is characterized by no sudden changes in the local velocity along the streamwise coordinate in all the flow conditions, as shown in Figure 6b,c. Because of these two characteristics, no stagnation zones form, and thus, no reduction of the working surface area is observed in the pure laminar regime.

An unsteady laminar regime establishes at 32 < Re_ds_ ≤ 128, characterized by the vortex shedding phenomenon according to the fluid dynamic simulations. No sharp transition of the Sherwood number dependency to the Reynolds number occurs between the pure laminar regime and the transitional laminar regime. Instead, the mass transfer progressively increases at increasing flow rate, leading to a dependence of the Sherwood number to the Reynolds number increased from 0.33 to 0.5 at increasing Re_ds_. When interpreting this growth in the range of 32 < Re_ds_ ≤ 128, the fitting of the data set leads for instance to a dependence of the Sherwood number on the Reynolds number increased to ∼0.45. A further increase could then be expected upon further increasing the Re_ds_, leading to the typical dependencies exhibited by flow around submerged objects in fully turbulent conditions, which establish at higher Re_ds_.^53,54^ It is worth noticing that a similar dependency arises for submerged cylinder in cross-flow in the unsteady laminar regime at 40 ≤ Re_D_ ≤ 4000, with Re_D_ being the Reynolds number referred to the diameter of the cylinder. In this regime, the Sherwood number depends on the Reynolds number raised to the power of 0.47.^49^

### 3.3 Effect of the Cell Size

**Figure 7:**
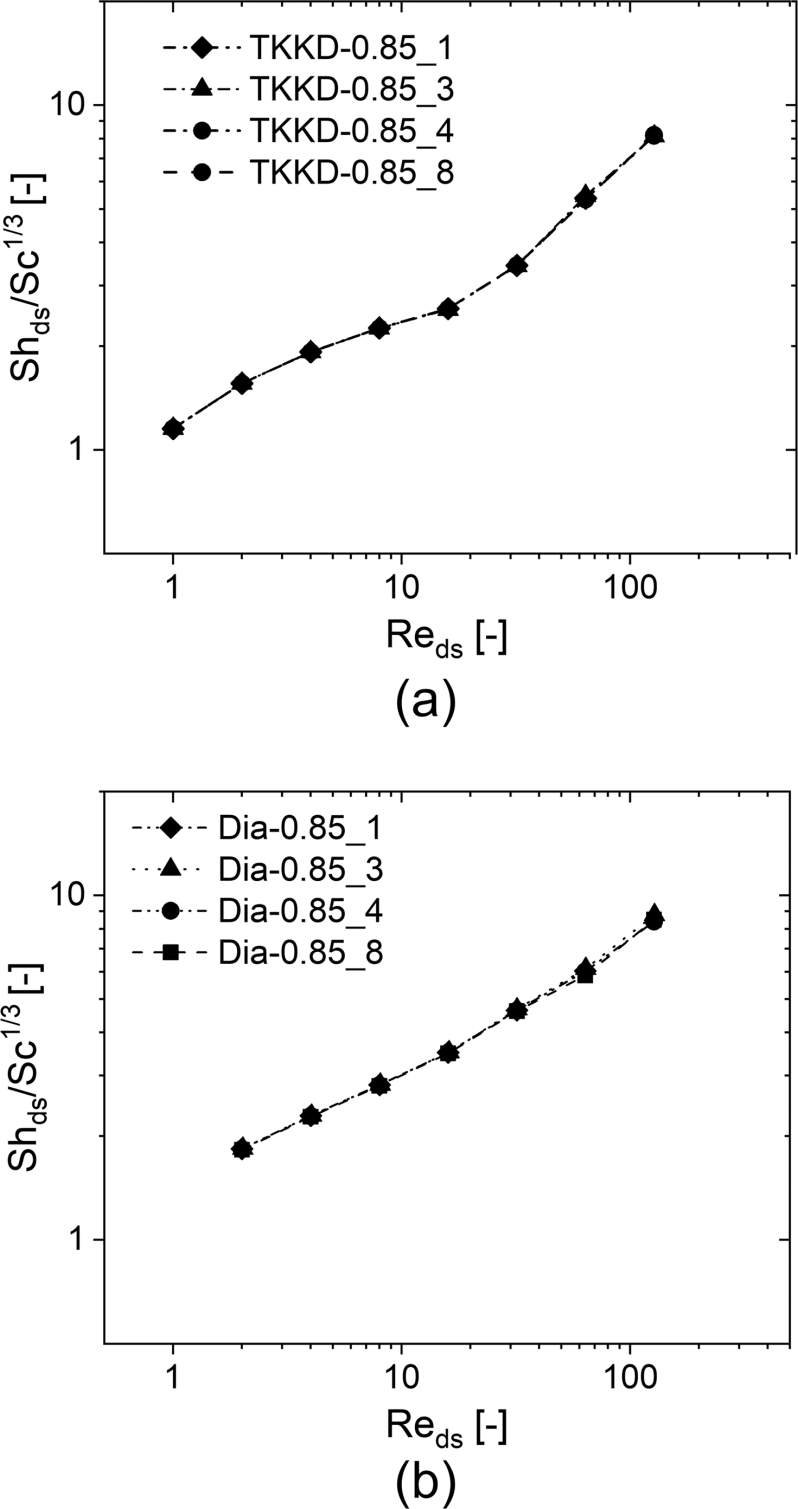
Sherwood numbers against the Reynolds numbers for (a) the TKKD unit cell POCS and (b) the Diamond unit cell POCS. The samples are characterized by porosity ε = 0.850, circular struts with constant cross-section and four different cell sizes (*d*_c_ = 1 mm Diamond, *d*_c_ = 3 mm triangles, *d*_c_ = 4 mm circles, *d*_c_ = 8 mm squares).

The dependence of the POCS external mass transfer properties on the cell size has been investigated by performing simulations on samples with different cell sizes for both the TKKD and the Diamond geometries. The considered cell sizes range between 1 and 8 mm. The samples geometrical parameters are reported in Table 2 and Table 3, while the simulation conditions are listed in Table 4. The simulations results are shown in Figure 7, where the Sherwood number is plotted against the Reynolds number for the TKKD (a) and the Diamond (b) samples. As the characteristic length, namely, the strut diameter, is linearly dependent to the cell size as discussed in Section 2.1, different fluid velocities were imposed in simulation to achieve the same Reynolds number at different cell sizes. In doing so, the Sherwood numbers are superimposed at the same Reynolds number regardless of the cell size. The same behavior has been reported for foams,^8,9^ whose Sherwood number is invariant with respect to the cell size at fixed porosity, when the strut diameter is adopted as the characteristic length. Hence, the effect of the cell size is already properly included in the dependency of the Sherwood number to the Reynolds number.

### 3.4 Effect of the Porosity

The effect of the porosity on the external mass transfer properties is assessed by considering six porosities ranging from 0.7 to 0.95 for both the TKKD and the Diamond unit cell POCS. Samples have a constant cell size *d*_c_ = 3 mm and constant circular strut section, to avoid any possible influence of these parameters.

**Figure 8:**
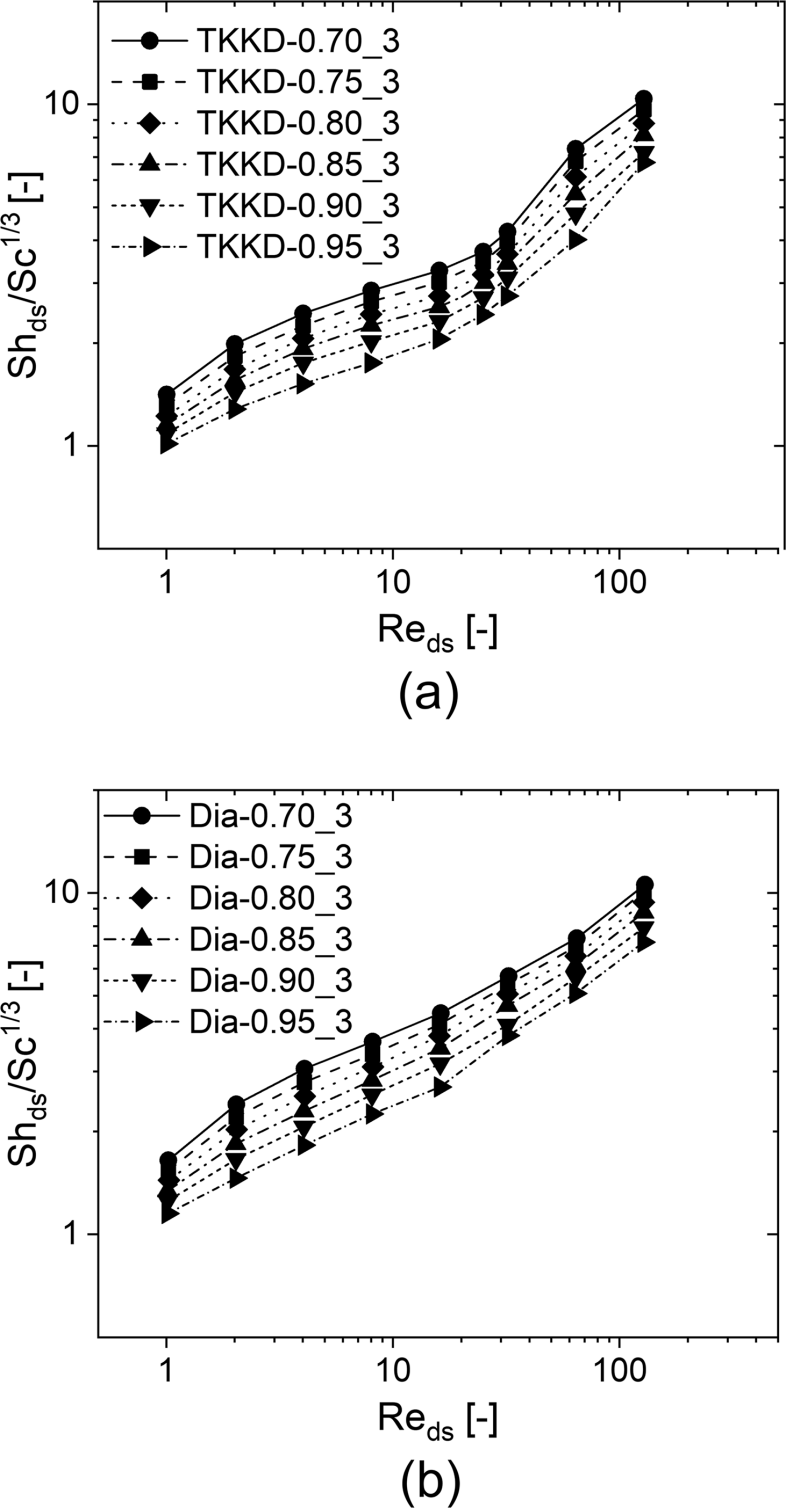
Sherwood numbers against the Reynolds numbers for (a) the TKKD unit cell POCS and (b) the Diamond unit cell POCS. The samples are characterized by cell size *d*_c_ = 3 mm, circular struts with constant cross-section, and six different porosities (ε = 0.700 circles, ε = 0.750 squares, ε = 0.800 diamonds, ε = 0.850 up-pointing triangles, ε = 0.900 down-pointing triangles, ε = 0.950 right-pointing triangles).

The simulation results are shown in Figure 8, where the Sherwood numbers are plotted against the Reynolds numbers for the TKKD samples (a) and the Diamond samples (b). The Sherwood number is observed to decrease with growing porosity at fixed Reynolds number. This behavior has been already reported for open-cell foams.^9^

**Figure 9:**
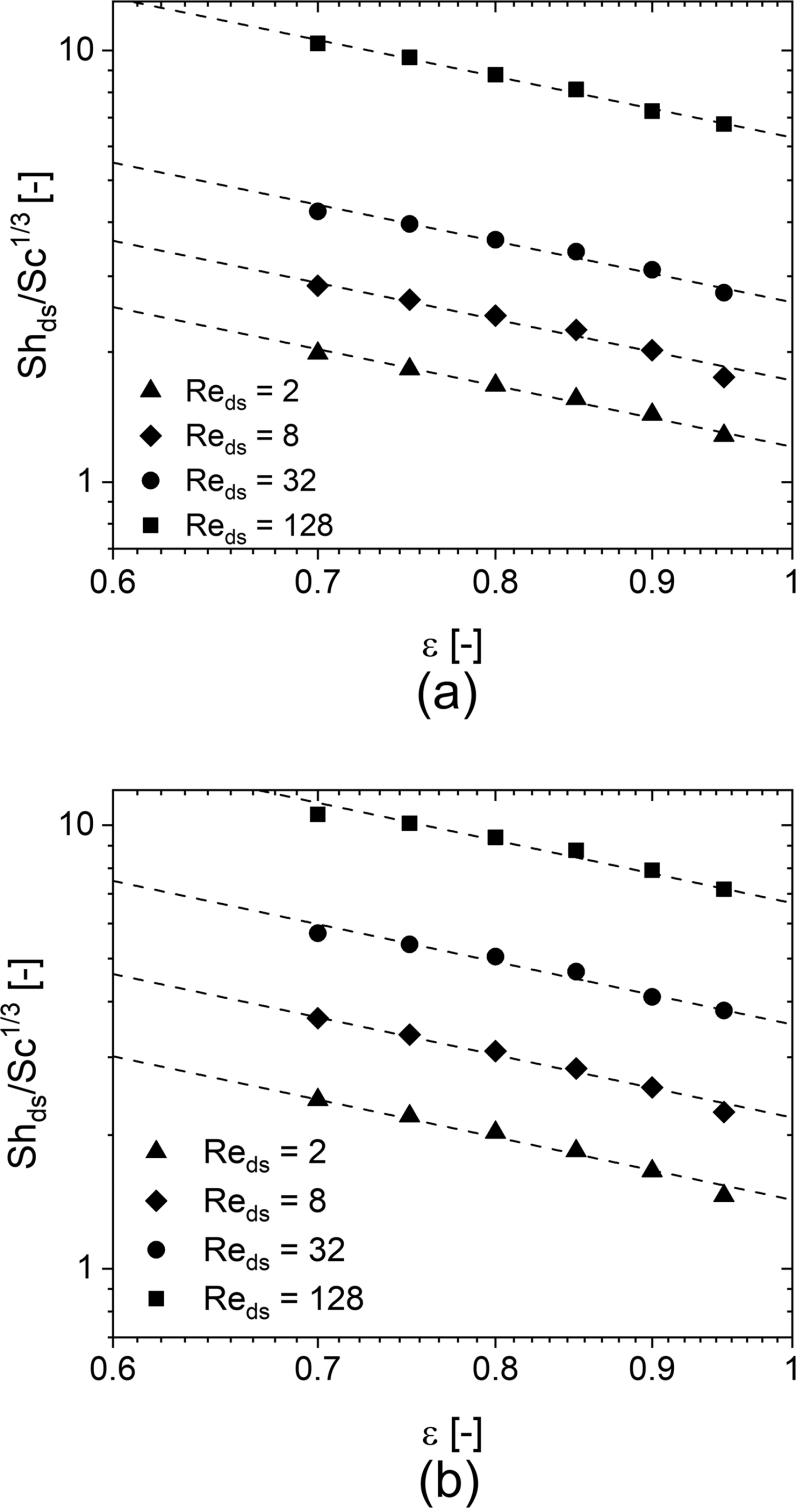
Sherwood number against the porosity for (a) the TKKD unit cell POCS and (b) the Diamond unit cell POCS. The dependency is shown at Re_ds_ = 2 (triangles), Re_ds_ = 8 (Diamond), Re_ds_ = 32 (circles), Re_ds_ = 128 (squares). Data are shown in log–log scale.

Figure 9 shows the Sherwood number against the porosity on a logarithmic scale for the two investigated POCS geometries at four chosen Reynolds numbers. Fitting an exponent of each data set of the Sherwood number at different Reynolds number leads to slopes ranging between −1.4 and −1.5 for both the TKKD and the Diamond geometries, whereas the best global data fit provides an empirical −1.5-order dependence of the Sherwood number on the porosity for both the geometries in all the flow conditions. It is worth noticing that such a dependency arises when considering the strut diameter as the characteristic length, which has been already proposed in our previous works for foams.^9,11^ In this regard, the Sherwood number evaluated in POCS shows a slightly weaker dependence on the porosity than the Sherwood number evaluated in foams,^9^ in which case the Sherwood number depends on the porosity raised to the power of −2. The slight difference in the functional dependence of the mass transfer coefficient on the porosity may be ascribed to the random configuration of the solid phase in foams, which results in a higher tortuosity of the fluid flow path. Consequently, a decrease in the porosity more strongly affects the gas–solid transfer rates in foams than in POCS.

### 3.5 Chilton–Colburn Analogy

Gas–solid heat transfer simulations are performed on samples with fixed porosity ε = 0.850 and cell size of *d*_c_ = 3 mm to cross-validate heat and mass transfer simulations and extend the previous findings to the modeling of gas–solid interphase heat transfer. Simulation conditions are reported in Table 4 and Table 5. It is worthwhile to emphasize that Nu_ds_ is evaluated at a prescribed wall temperature; hence, it is possible to disregard the heat conduction mechanism in the solid due to temperature inhomogeneity, which may be significant in real applications especially at low Re_ds_ and which can be accounted by different models hereby not discussed.

**Figure 10:**
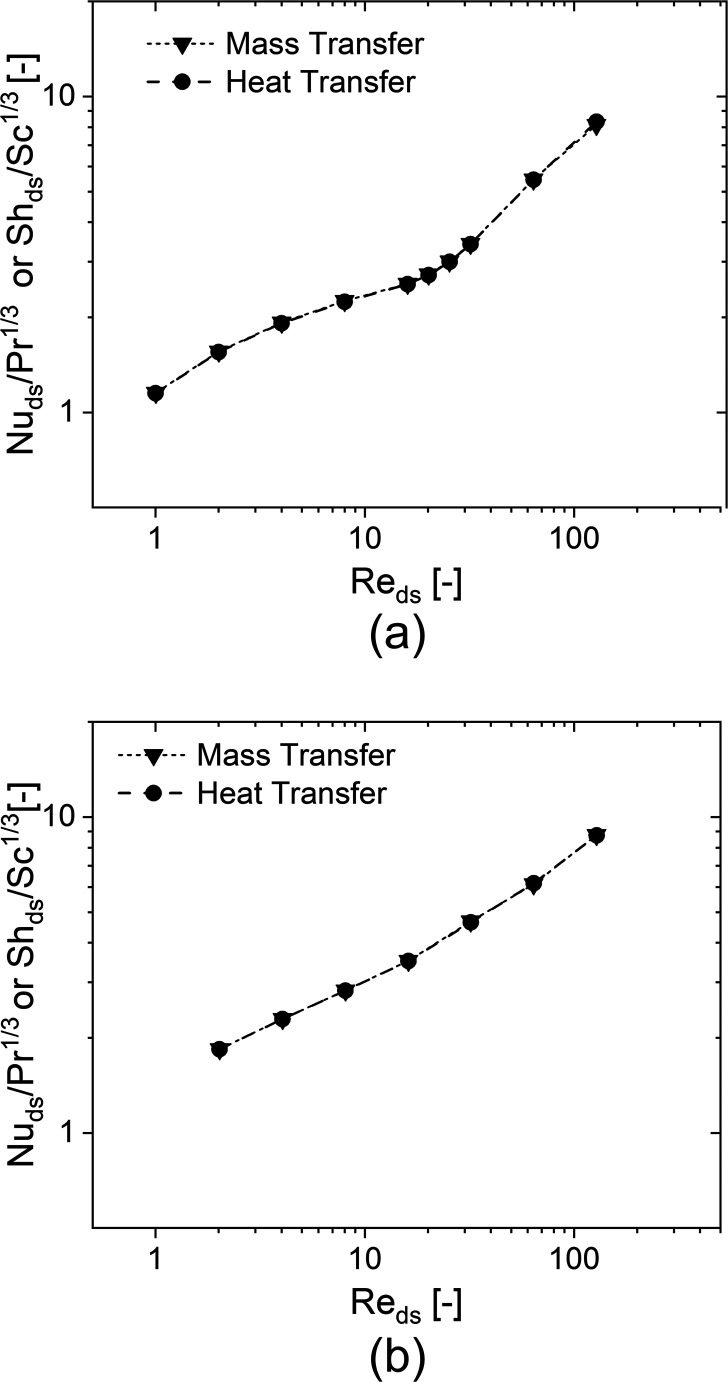
Sherwood numbers and Nusselt numbers against the Reynolds numbers for (a) the TKKD unit cell POCS and (b) the Diamond unit cell POCS. The sample features porosity ε = 0.850, cell size *d*_c_ = 3 mm, and circular struts with constant cross-section.

Figure 10 shows the Sherwood number and the Nusselt number plotted against the Reynolds number for (a) the TKKD and (b) the Diamond samples. The Nusselt numbers are superimposed to the Sherwood numbers in all the considered flow conditions, proving the applicability of the Chilton–Colburn analogy for heat and mass transfer in POCS. Hence, the results from the previous analyses of gas–solid interphase mass transfer can equally be applied in terms of heat transfer. Along the same lines, the following discussion and the derived correlations will hold for both external heat and mass transfer.

## 4 Discussion

In this section, we develop descriptive engineering correlations to estimate the transport properties of POCS as a function of their geometrical properties and on the basis of the identified flow regimes. Finally, POCS performances in the mass transfer limited regime will be compared to those of other structured supports.

### 4.1 Assessment of the Characteristic Length

As discussed in Section 2.1, two independent geometrical parameters are required to define POCS geometries. Following the geometrical characterization performed in this work, the cell size and the porosity have been chosen as design parameters, and any other geometrical feature can be evaluated by geometrical models. Hence, multiple choices are possible in the definition of a characteristic length, which could be any one of the estimated geometrical features.

Herein, the strut diameter has been selected as characteristic length, consistently with our previous works on open-cell foams.^9,11^ The strut diameter is proposed in analogy with the tube diameter for heat transfer in tube banks and with other common choices in the literature to characterize the heat transfer in flow around submerged objects. Moreover, the strut diameter has proven to be the most suitable choice in catching the transition between the Darcy and the post-Forchheimer flow regimes at the typical Reynolds numbers for cylinders in crossflow for both foams and POCS, that is, roughly at Re_ds_ ≈ 40. However, POCS complex solid matrices induce the fluid to flow in tortuous paths with local acceleration and deceleration, as shown by Figure 5 and Figure 6, inevitably introducing local changes of velocity. Therefore, different from tube banks and cylinders in cross-flow, the transition occurs in a broader range of Reynolds numbers, rather than at a given Re number like in the case of other systems.

Other geometrical quantities have been proposed as characteristic length in the literature. The interpretation of the current results relative to heat and mass transfer in POCS according to the hereby discussed characteristic lengths and the extensive discussion is reported in the Supporting Information. Conclusively, we consider the strut diameter as the most physically sound characteristic length scale, enabling us to catch the transition between the different flow regimes at the same characteristic Reynolds number of tube banks. Furthermore, the strut diameter represents a key parameter in POCS manufacturing by 3D-printing, being the minimum printable detail. The strut diameter as the characteristic length is therefore two-fold significant: it readily provides the manufacturability of the catalyst support and conveys the engineering information related to the catalytic reactor design.

### 4.2 Heat and Mass Transfer Correlation

The present numerical study has revealed that the convection mechanism depends on the flow conditions, the porosity, and the cell shape of POCS, whereas by adopting a correct characteristic length, it is possible to include the effect of the cell size in the Sh-Re (or Nu-Re) dependency. Consequently, we propose an engineering correlation for each one of the two considered unit cells to express the Sherwood or Nusselt numbers as a function of the Reynolds number, the Schmidt, or the Prandtl number and the porosity. According to the numerical simulations, an empirical dependence of the Sherwood number on the porosity raised to the power of −1.5 is derived for the two considered geometries. The Sherwood/Nusselt number is assumed to be dependent on the Schmidt/Prandtl number raised to the power of 1/3 according to the boundary layer theory.^43^ In this view, correlations have been obtained for gas–solid systems in the range of Sc (or Pr) around 1. To account for the dependence on the flow conditions, two distinct approaches are proposed for the two considered unit cells.

#### 4.2.1 TKKD Unit Cell

As discussed in Section 3.2, the dimensionless mass transfer coefficient of the TKKD unit cell exhibits three different dependencies on the Reynolds number as a function of the flow regime. Therefore, the correlations assume the functional form in eq 8.
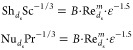
8

The values of the exponent *m* established in Section 3.2 are retained for the determined flow regimes. The coefficient *B* is then estimated from a nonlinear regression of the numerical results to minimize the residual sum of squares between the numerical results and the model predictions.

The parameters *B* and *m* are listed in Table 6. The coefficients provide a continuous function in the considered Re_*d*_s__ range. It is worth noticing that the values of *B* = 0.924 and *m* = 0.33 were obtained in the creeping viscous flow in the range 1 ≤ Re_*d*_s__ ≤ 4. These values are comparable to the one reported by Hilpert^49^ for a cylinder submerged in fluid flow at around the same Re, i.e., *B*′ = 0.989 and *m* = 0.330. The comparison between POCS and cylinders demonstrates the strong analogy when considering the strut diameter as the characteristic length. Along the same lines, values of *B* = 1.061 and *m* = 0.23 are obtained in the pure laminar regime as an effect of the struts mutual hiding/masking discussed in Section 3.2, which is comparable to the same condition for a cylinder submerged in fluid flow.^49^ In the unsteady laminar regime, values of *B* = 0.257 and *m* = 0.67 are obtained. The occurrence of the vortex shedding phenomenon causes a sudden transition from the pure laminar to the unsteady laminar regime, thus inducing a steeper increase of the gas–solid interphase transfer rates in this regime. As discussed in Section 3.2, the observed dependency 0.67 compensates the weak dependency 0.23 observed in the pure laminar regime. In this view, the exponent 0.67 is slightly higher than the dependency 0.45 observed in the case of the Diamond unit cell in the unsteady laminar regime (see Section 3.2).

**Table 6:** Coefficients of Equation 8

Re_*d*_s__ range	*B*	*m*
1 ≤ Re_*d*_s__ ≤ 4	0.924	0.33
4 < Re_*d*_s__ ≤ 25	1.061	0.23
25 < Re_*d*_s__ ≤ 128	0.257	0.67

The correlation (8) provides the best representation of the external transport properties of the TKKD unit cell based on the numerical simulations.

**Figure 11:**
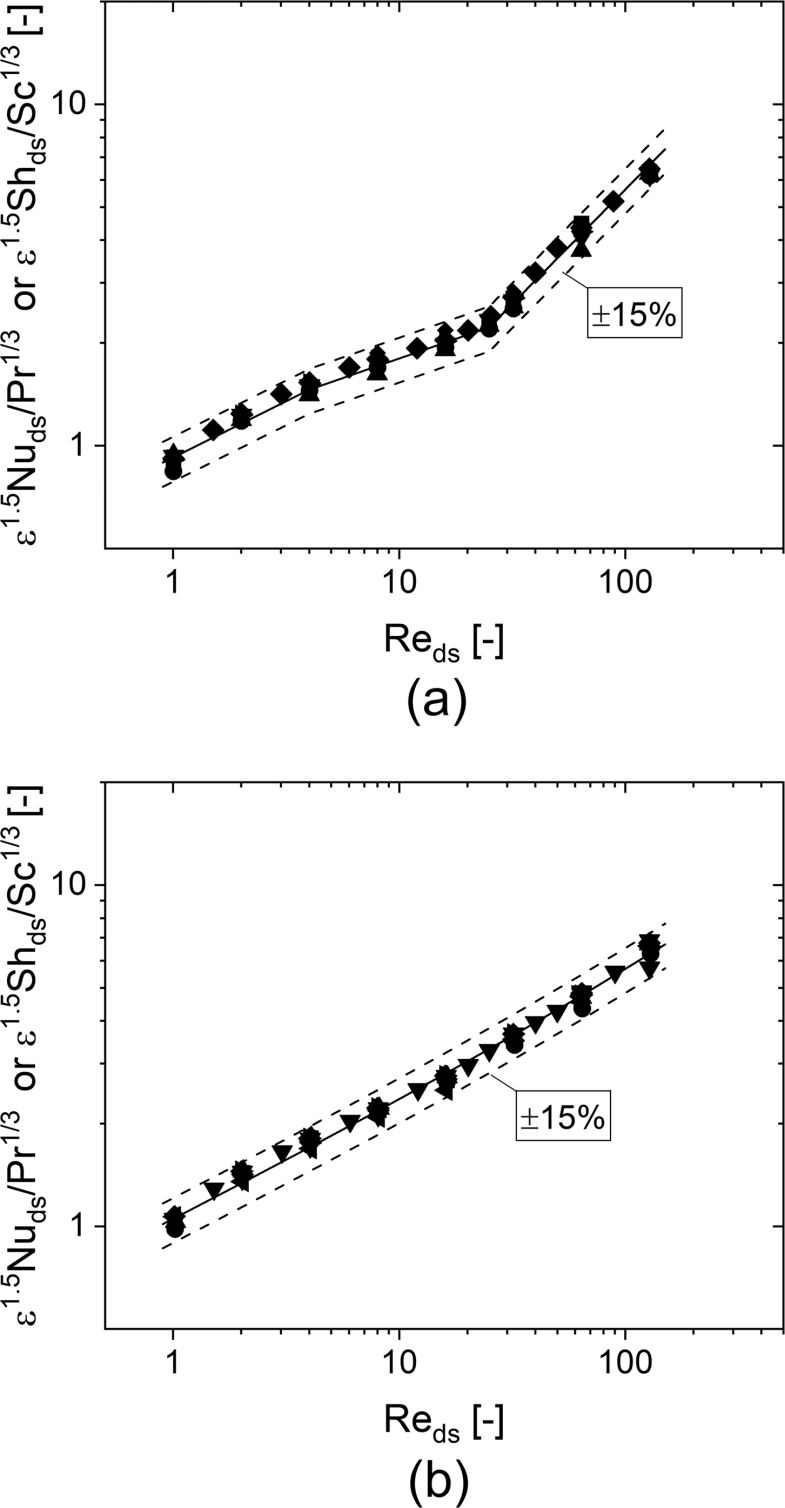
Sherwood number as a function of the Reynolds number for the CFD simulation along with the proposed mass transfer correlation for (a) the TKKD unit cell and (b) the Diamond unit cell.

Figure 11a reports the obtained correlation along with the simulations data, showing a good agreement with deviations below ±15% between the predictions and the data. The applicability ranges of the derived correlation are 1 ≤ Re_*d*_s__ ≤ 128, 0.75 ≤ Sc (or Pr) ≤ 1.5, 0.7 ≤ ε ≤ 0.95 and 1 ≤ *d*_c_ ≤ 8 mm.

#### 4.2.2 Diamond Unit Cell

To model the convective transport properties of the Diamond unit cell, the effect of the flow conditions is accounted by using two asymptotic contributions, related to the creeping flow and to the post-Forchheimer regimes, as proposed in the context of generalized fibrous media by Reichelt et al.^55^ and employed in our previous work for open-cell foams.^9^ The fully laminar regime is modeled by using a functional dependency of the Sherwood number on the Reynolds number raised to the power of 1/3. This choice relies on the Diamond unit cell behavior discussed in Section 3.2. Furthermore, this behavior is also associated with creeping viscous flow past cylinders and arrays of cylinders.^49,50^ The second asymptotic contribution is provided by a dependence of the dimensionless transfer coefficient on the Reynolds number raised to 0.8, which is reported for fully turbulent flow in tube bundles^56^ and for a single cylinder in cross-flow in the turbulent flow as well.^49^ It should be noted that the superposition of the asymptotic contributions related to the creeping flow and the post-Forchheimer regimes leaded to an apparent dependency of the Sherwood number on the Reynolds number raised to the power of 0.45 in the unsteady laminar regime investigated in this work for 32 ≤ Re_*d*_s__ ≤ 128, as shown in Section 3.2. Consequently, the correlation takes the form:
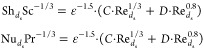
9

The numerical coefficients are evaluated from the regression of the numerical data. Hence, the following correlation gives the best description of dimensionless transport coefficients for Diamond unit cell POCS:
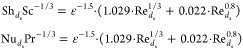
10

It is worth noticing that values of *C* = 1.029 and *D* = 0.022 have been obtained for the creeping flow and the fully turbulent asymptotic contributions. The values are comparable to the one obtained for a single cylinder submerged in cross-flow in the two regimes.^49^ Indeed, for a single cylinder *C* = 0.989 for the creeping viscous flow at 0.4 ≤ Re_*D*_ ≤ 4 and *D* = 0.027 for the turbulent flow at 4 × 10^4^ ≤ Re_*D*_ ≤ 4 × 10^5^, with Re_*D*_ being the Reynolds number referred to the cylinder diameter. The simulations data are plotted along with the correlation given by eq 10 in Figure 11b: a good agreement is observed, with deviations below ±15% between the predictions and the data. The applicability ranges of the derived correlation are 1 ≤ Re_*d*_s__ ≤ 128, 0.75 ≤ Sc (or Pr) ≤ 1.5, 0.7 ≤ ε ≤ 0.95 and 1 ≤ *d*_c_ ≤ 8 mm.

### 4.3 Performance Comparison of Structured Supports

POCS are envisioned as potential candidates for enhanced catalyst supports with intensified external transfer rates. This aspect is pivotal, for example, in aftertreatment applications, where space and mass limitations are coupled with the requirement of lower emissions, and in chemical syntheses, where the heat management is often crucial. The interphase heat and mass transfer in POCS is hereby evaluated by comparison of the volumetric mass transfer coefficient, defined as
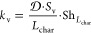
11

Combining eqs 4 and 11, the volumetric mass transfer coefficient can be expressed as
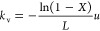
12

In the mass transfer limited regime, for a given application (given support length *L*), the mass transfer limited conversion increases on increasing the volumetric mass transfer coefficient at fixed fluid velocity or residence time. As expressed by eq 11, the volumetric mass transfer coefficient depends on the geometrical features of the support, the fluid thermophysical parameters, and the flow rate.

In this view, the supports performance is examined by considering CO combustion in air at 573 K and 1 atm as the test reaction, which is typical for environmental catalysis applications. POCS geometrical features are considered to comply with the current 3D-printing manufacturing limitations; therefore, the strut diameter is chosen as the design parameter being the structure minimum printable detail.^57,58^ In addition, porosities in the range ε = 0.7–0.95, typical of a structured catalyst support, are considered.

**Figure 12:**
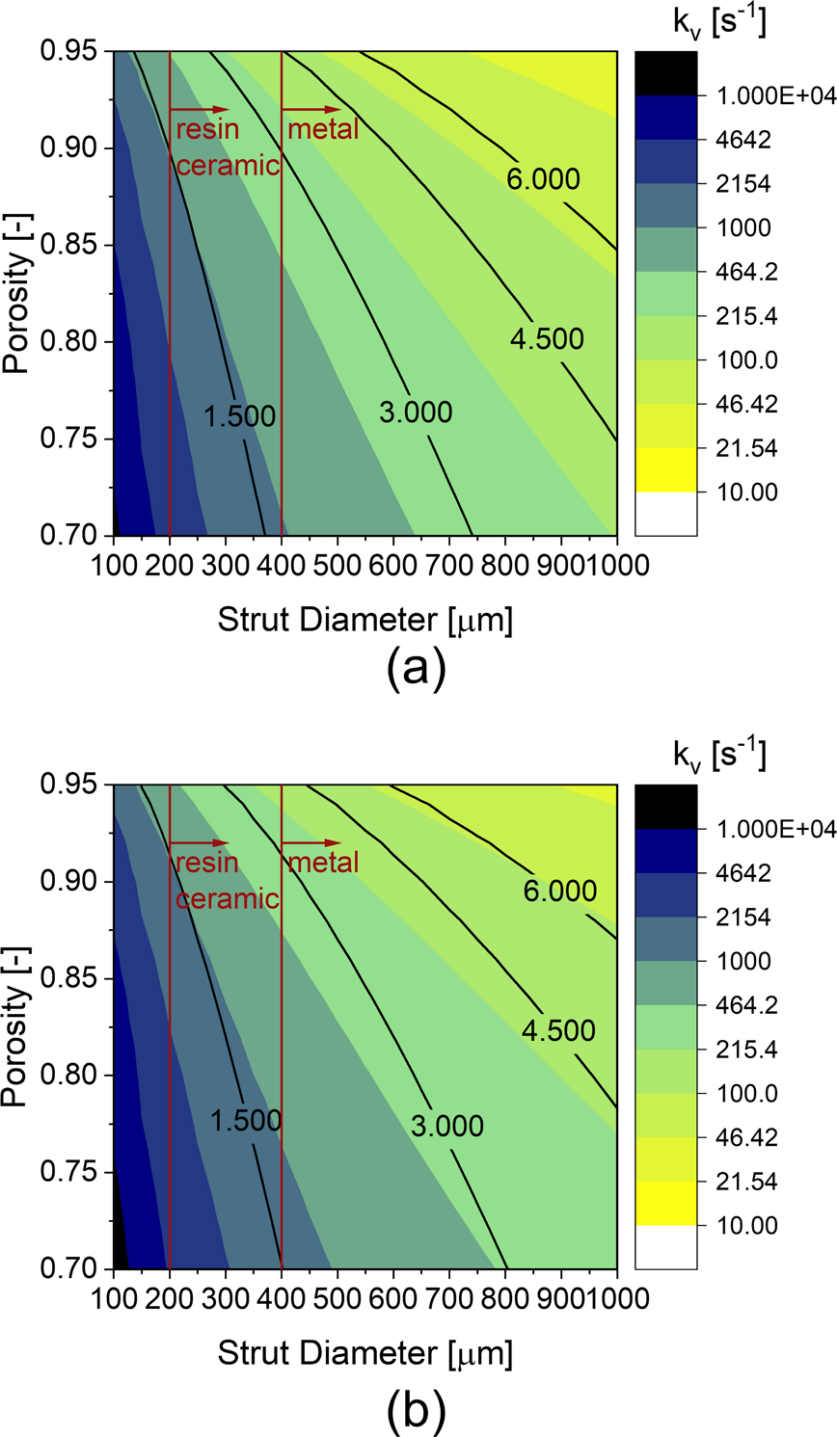
Volumetric mass transfer coefficient *k*_v_ as a function of the porosity and the strut diameter at prescribed fluid velocity u = 1 m/s for (a) the TKKD and (b) the Diamond unit cells (color palette is in logarithmic scale). The corresponding cell sizes are reported in millimeters as black lines, while the limits imposed by the current manufacturing technologies are plotted as vertical red lines.

Figure 12 shows the volumetric mass transfer coefficients of the TKKD (a) and the Diamond (b) unit cells plotted against the strut diameter at different porosities at a fixed fluid velocity of 1 m s^–1^, corresponding to a specific mass flow rate of 0.6 kg m^–2^ s^–1^. The volumetric mass transfer coefficient increases at decreasing porosity and strut diameter. Indeed, as expressed by eq 11, the volumetric mass transfer coefficient is inversely proportional to the strut diameter and linearly dependent on the specific surface area, which increases with decreasing strut diameter and porosity (see Figure 2).

**Figure 13:**
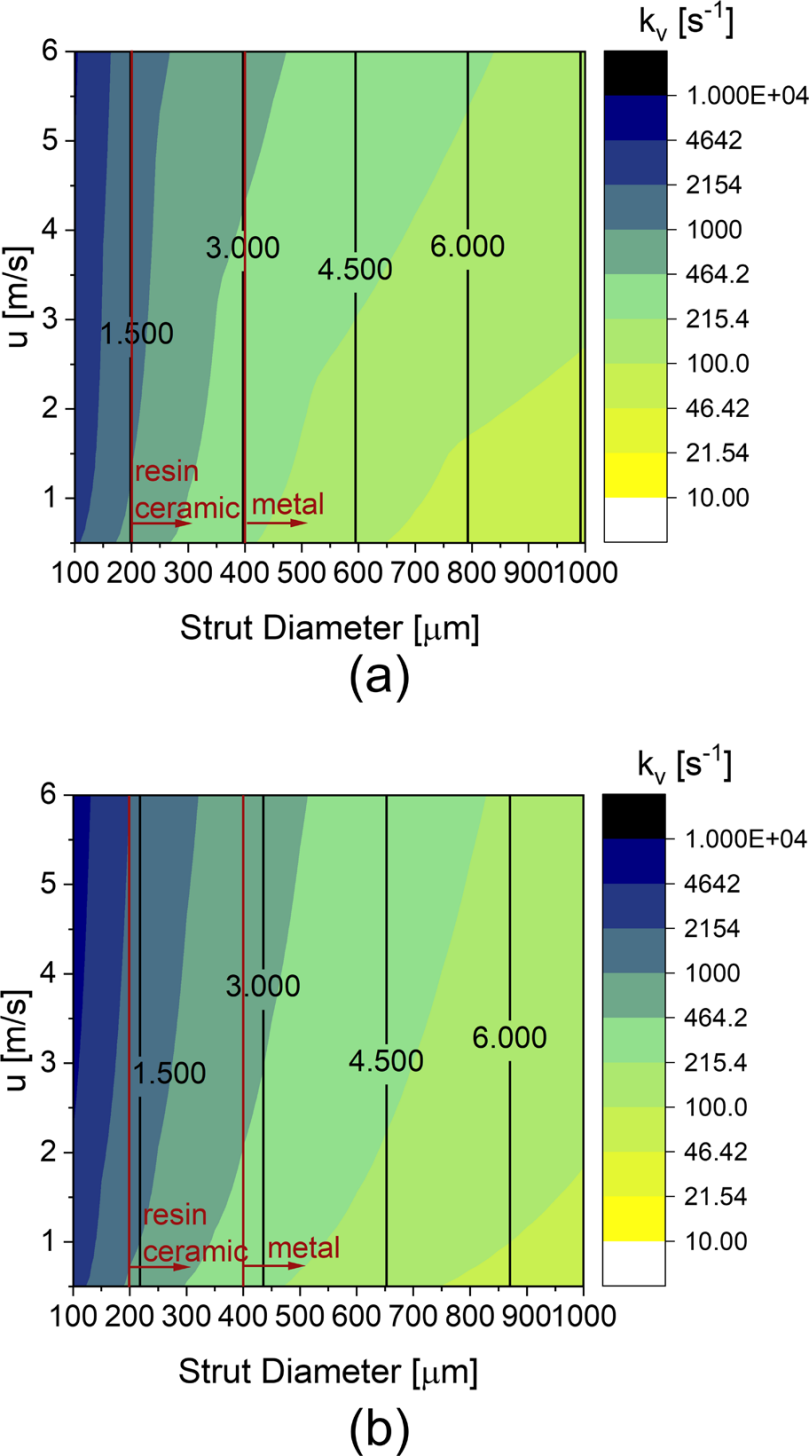
Volumetric mass transfer coefficient *k*_v_ as a function of the strut diameter and the fluid velocity at prescribed porosity ε = 0.9 for (a) the TKKD and (b) the Diamond unit cells (color palette is in logarithmic scale). The corresponding cell sizes are reported in millimeters as black lines, while the limits imposed by the current manufacturing technologies are plotted as vertical red lines.

Figure 13 shows the volumetric mass transfer coefficients of (a) the TKKD and (b) the Diamond unit cells as a function of the velocity and the strut diameter at fixed porosity ε = 0.9. The volumetric mass transfer coefficient increases at increasing fluid velocity, as the associated Sherwood number grows with Reynolds number. The interphase mass transfer increases with decreasing strut diameter and with increasing flow rate. Thus, miniaturization of the structured supports is crucial in view of chemical reactors intensification. POCS manufacturing, provided by 3D-printing, is currently limited by the existing technologies, as highlighted in Figure 12 and Figure 13. Figure 12 and Figure 13 show POCS promising potential for the intensification of structured catalytic reactors.

As a benchmark, a state-of-the-art square channel honeycomb characterized by 900 channels per square inch (CPSI) and a porosity of 0.85 can be considered. The honeycomb is characterized by constant Sherwood number, function of the channel geometry only when neglecting entrance effects.^40^ In the considered conditions, the honeycomb offers a volumetric mass transfer coefficient *k*_v_ = 1100 s^–1^ in any flow condition, corresponding to a mass transfer limited conversion *X* = 67% considering a residence time equal to τ = 0.001 s. Figure 12 shows that such a performance can be matched by a Diamond unit cell POCS with porosity ε = 0.9, cell size *d*_c_ = 1.500 mm and strut diameter *d*_s_ = 0.200 μm at fluid velocity u = 1 m s^–1^ (specific mass flow rate *G* = 0.6 kg m^–2^ s^–1^, Re_*d*_s__ = 4). Contrarily to the honeycomb monolith, POCS Sherwood numbers and thus volumetric mass transfer coefficients increase with increasing fluid velocity (Figure 13). The considered Diamond unit cell POCS offers a volumetric mass transfer coefficient *k*_v_ = 2000 s^–1^ at fluid velocity u = 5 m s^–1^ (specific mass flow rate G = 3 kg m^–2^ s^–1^, Re_ds_ = 20), leading to a mass transfer limited conversion X = 86% with a residence time equal to τ = 0.001 s. The Diamond performances can be compared to other cellular materials, such as foams. Foams are characterized by different manufacturing techniques,^59^ with lower limits in miniaturization when compared to POCS. In this view, foams with cell size in the range of 1 mm offer intense gas–solid transfer rates.^9^ On the other hand, upon reduction of the pores size, higher pressure loss may lead to unsatisfactory performances of the substrate.^11^ Thus, a foam with porosity ε = 0.9 and average strut diameter d_s,avg_ = 200 μm can be considered to match the characteristics of the considered Diamond, and reasonably compare their performance. The foam properties are evaluated on the basis of the information provided by Bracconi et al.^9^ In the discussed flow conditions, namely at fluid velocities u = 1 m s^–1^ and 5 m s^–1^, the foam offers a volumetric mass transfer coefficient *k*_v_ = 670 s^–1^ and 1290 s^–1^ leading to a mass transfer limited conversion X = 49% and 73%, respectively with a residence time equal to τ = 0.001 s. The foam offers significantly poorer performances than the Diamond, and the target conversion given by the state-of-the-art honeycomb is reached at fluid velocity *u* ≥ 3.5 m s^–1^. Such discrepancy may be ascribed to the lower specific surface area of foams with respect to POCS at the same strut diameter (∼25% lower), although the still comparable cell size (see Table 7). This may be due to the parabolic struts of foams, which offer lower surface area than POCS circular struts with constant cross-section.

**Table 7:** Geometrical Properties of the Compared Supports

sample	*d*_c_ or *d*_channel_ [mm]	ε or OFA [-]	*S*_v_ [m^–1^]	*L*_char_ [mm]
TKKD	1.513	0.9	1813	0.200
diamond	1.379	0.9	1836	0.200
foam	1.583	0.9	1377	0.200
honeycomb	0.847	0.85	4355	0.781

Finally, we compare the overall performances of POCS with those of open-cell foams and honeycomb monoliths. The geometrical properties of the considered supports are listed in Table 7. The state-of-the-art square channel honeycomb characterized by 900 channels per square inch (CPSI), and an open frontal area (OFA) of 0.85 is considered. POCS are considered with porosity ε = 0.9 and strut diameter *d*_s_ = 200 μm, compliant with the current limitations of ceramic POCS manufacture by 3D-printing.^58^ The foam with circular strut, porosity ε = 0.9, and average strut diameter *d*_s,avg_ = 200 μm is considered to match the POCS geometrical features.

**Figure 14:**
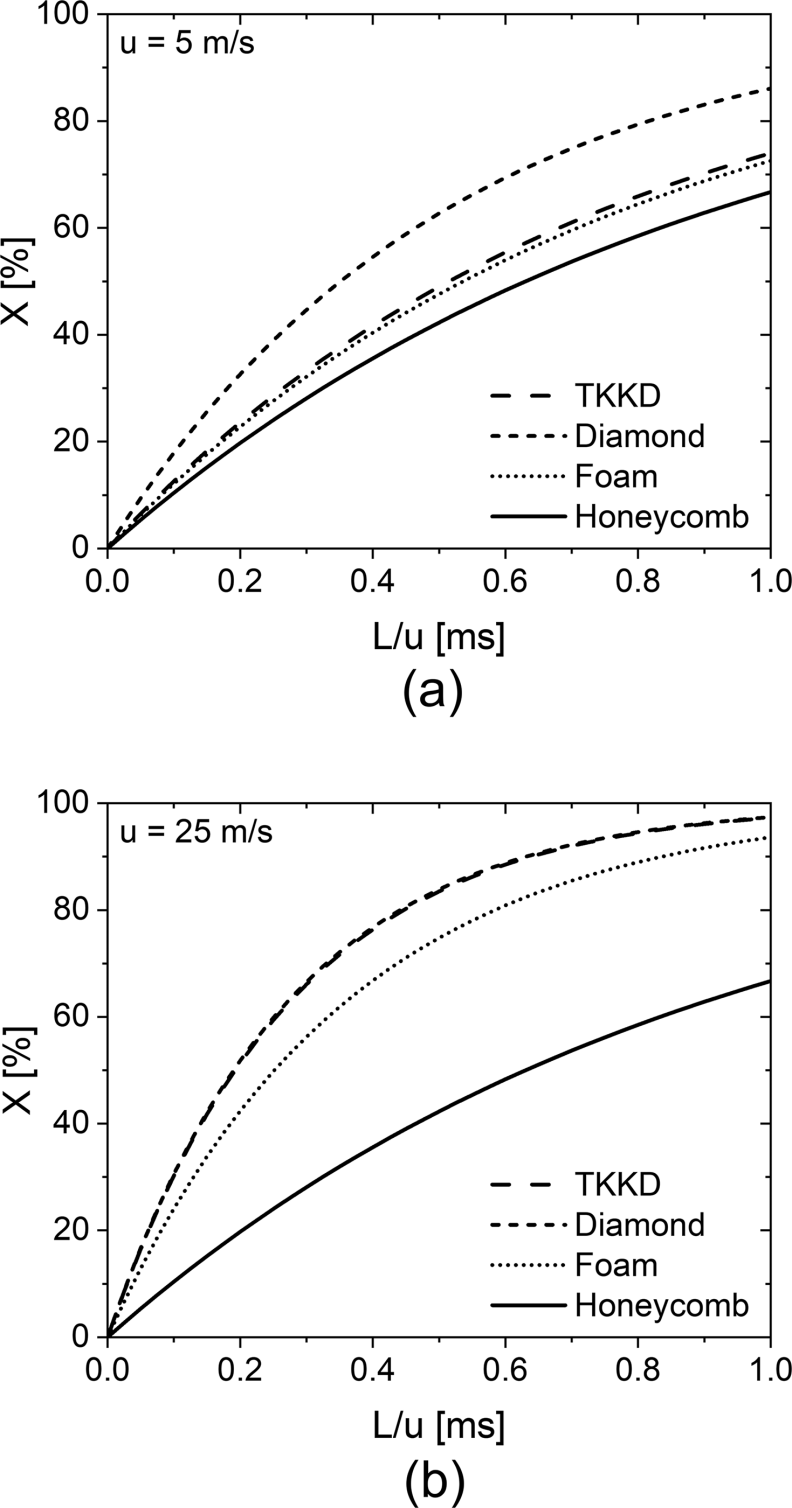
Conversion versus residence time for POCS (dashed lines), foams (dotted lines) and honeycombs (solid lines) at fluid velocities (a) *u* = 5 m/s and (b) *u* = 25 m/s.

Figure 14 shows the mass transfer limited conversion against the residence time for the four considered supports at fluid velocities of 5–25 m/s, corresponding to a Re_*d*_s__ = ∼20–100 for the POCS and the foam, which are representative of possible operating conditions in real applications. At low velocity, cellular materials outperform the state-of-the-art honeycomb substrate; moreover, POCS offer higher conversion than the considered foam, with the Diamond unit cell POCS offering the best performance. At high velocity, the difference between cellular materials and the state-of-the-art honeycomb substrate widens, as the interphase mass transfer in cellular materials is enhanced at increasing flow rate; the TKKD and the Diamond POCS exhibit equivalent performance and provide higher conversions than the foams.

In complement to this analysis, it should be remarked that the application of structured catalyst supports at the industrial scale is generally ruled by different properties, too, which must be considered for the optimal reactor design. As an example, their use in aftertreatment devices is governed by the trade-off between mass transfer and pressure drop, represented for instance by the Merit Index introduced by Giani et al.^8^ Hence, further studies are required to estimate POCS overall performance taking also into account the pressure drop in POCS^14,52^ and provide a systematic evaluation of the Merit Index in the conditions of real applications.

## 5 Conclusions

In this work, we have proposed a CFD-based systematic investigation of the interphase gas–solid heat and mass transport in periodic open cellular structures in fully developed flow conditions. The Diamond unit cell and tetrakaidekahedral (TKKD) unit cell have been examined. The hierarchical approach has been exploited for the analysis of the structure behavior in fully developed flow conditions, which were modeled assuming periodic boundary conditions. CFD simulations on virtually constructed POCS have been exploited to model the effects of the flow conditions and of the geometrical features of POCS on the transport rates. The inspection in the flow field in POCS has revealed that distinct flow regimes occur in the two different unit cells, which are strictly related to their morphological features. In the case of the TKKD unit cell, a creeping viscous flow arises at low Reynolds number at Re_*d*_s__ ≤ 4. In the subsequent laminar regime at 4 ≤ Re_*d*_s__ ≤ 25, a reduced dependence of the interphase transfer rate on the growing flow rate is observed, and this phenomenon is ascribed to the mutual hiding/masking of struts lined up in the flow direction. At higher flow rates at Re_*d*_s__ ≥ 25, an unsteady laminar regime characterized by the vortex shedding phenomenon occurs. In this regime, the interphase transfer is promoted by the vortices, and a steeper increase of the interphase transfer rate is observed. In the case of the Diamond unit cell, a pure laminar regime arises at moderate Reynolds number (1 ≤ Re_*d*_s__ ≤ 32). Contrarily to the TKKD unit cell, no detrimental effect due to struts mutual hiding/masking is observed on the interphase transfer rate. An unsteady laminar regime occurs as well for the Diamond unit cell at Re_*d*_s__ ≥ 32. The interphase transfer rate in the Diamond unit cell is thus observed to progressively increase with the increase of the flow rate both in the pure laminar and in the unsteady laminar regime. When considering the strut diameter as the characteristic length, the transition between the flow regimes is found to happen for both the unit cells at around the same literature value of critical Reynolds number for flow around submerged objects, which supports the choice of the strut diameter as the characteristic length. Conversely, when prescribing the porosity and the cell size as POCS design parameters, the dimensionless mass transfer coefficient of ideal POCS with circular struts is function of the porosity and of the flow conditions only. An average dependence of the Sherwood number on the porosity raised to −1.5 was found. The validity of the analogy between intraphase mass and heat transfer was proven for POCS in the pure laminar and unsteady laminar regimes. Consequently, the results discussed in the mass transfer analysis apply as well in the terms of Nusselt number, and thus the results can be equally applied to model the fluid–solid heat transfer.

Two distinct approaches have been proposed to model the dimensionless transport coefficient of the two unit cells, in order to account for the discussed effects of the flow conditions. Consistently, engineering correlations able to describe the heat and mass transfer coefficient as a function of the geometrical properties and the flow conditions have been derived. The simulations data are accurately described with deviations less than 15%. The derived correlations were exploited to provide simplified tools for the immediate evaluation of the transport coefficient as a function of the fluid dynamic conditions and POCS geometrical parameters of design. POCS miniaturization is beneficial to achieve higher gas–solid transfer rates due to higher specific surface area. In this view, further development of 3D-printing techniques is desirable. Nonetheless, the gas–solid transport coefficient of cellular materials is shown to increase at increasing working fluid velocity, in contrast to the state-of-the-art structured support for catalytic applications, i.e., the honeycomb monolith, whose transport coefficient is solely determined by its geometrical features. Owing to this benefit, cellular materials in the current range of manufacturability already outperform the established technology in term of gas–solid transfer rates in a broad range of working conditions. Additionally, POCS prove to offer the highest transfer rates among the considered cellular materials when compared at same porosity and strut diameter. In this view, the Diamond lattice offers the best performance. In conclusion, the simplified tools allow for the design of novel catalytic reactors with innovative structured supports based on 3D printed POCS.

## Supporting Information Available

TKKD geometrical model; mesh generation; periodic boundary conditions for 3D flows; mesh convergence analysis; assessment of the characteristic length (extended) (PDF)

## Dedication

This paper is dedicated to Prof. Giuseppe Storti on the occasion of his 65th birthday.
