# Supplementary material for: A Fundamental Investigation of Gas/Solid Heat and Mass Transfer in Structured Catalysts Based on Periodic Open Cellular Structures (POCS)
Source: Ind Eng Chem Res. 2021 Jun 15;60(29):10522–38. doi: 10.1021/acs.iecr.1c00215 (PMC8323103; doi:10.1021/acs.iecr.1c00215)
Supplement: Supplementary file 1 — ie1c00215_si_001.pdf [file ie1c00215_si_001.pdf]

Supporting Information for:

A fundamental investigation of gas/solid heat and mass transfer in structured catalysts based on Periodic Open Cellular Structures (POCS)

*Claudio Ferroni[a], Mauro Bracconi[a], Matteo Ambrosetti[a], Matteo Maestri[a], Gianpiero Groppi[a], Enrico Tronconi[a]**

[a]Laboratory of Catalysis and Catalytic Processes, Dipartimento di Energia, Politecnico di Milano

via La Masa 34, 20156 Milano, Italy

*to whom correspondence should be addressed:

enrico.tronconi@polimi.it

Table of contents

1. TKKD geometrical model S3

2. Mesh generation S6

3. Periodic boundary conditions for 3D flows S8

4. Mesh convergence analysis S12

5. Assessment of the characteristic length (extended) S14

References S20

1. TKKD geometrical model

Ambrosetti et al.[1] developed a geometrical model for open-cell foams on the basis of the TKKD unit cell with struts with variable cross section between the node and the middle of the ligament. The approach can be extended to model the ideal TKKD unit cell with circular struts with constant cross section. The TKKD unit cell porosity and specific surface area can be expressed as follows:

$$\varepsilon = 1 - \frac{6V_{node} + 12V_{strut}}{V_{cell}} \quad \text{(S1)}$$

$$S_v = 1 - \frac{6S_{node} + 12S_{strut}}{V_{cell}} \quad \text{(S2)}$$

where V_{node} and V_{strut} indicate the volume of a single node and of a single strut respectively and, likewise, S_{node} and S_{strut} indicate the exposed area of a node and of a strut. It is worth to emphasize that the struts junction in the node must be modelled in detail.

In each node, four struts converge, and the angle between the struts corresponds to the tetrahedral or Plateau angle:

$$\gamma = arccos\left(-\frac{1}{3}\right) \cong 109.47° \quad \text{(S3)}$$

The node volume is computed by considering the node as a sphere cut by the four struts. The node diameter can be expressed as a function of the strut diameter and of the tetrahedral angle according to Eq. (S4):

$$d_n = \frac{d_s}{sin\left(\frac{\gamma}{2}\right)} = \sqrt{\frac{3}{2}}d_s \quad \text{(S4)}$$

The struts remove from the node four spherical caps with height:

$$h = \frac{d_n}{2}\left(1 - cos\left(\frac{\gamma}{2}\right)\right) = \frac{d_s}{2\sqrt{2}}\left(\sqrt{3} - 1\right) \quad \text{(S5)}$$

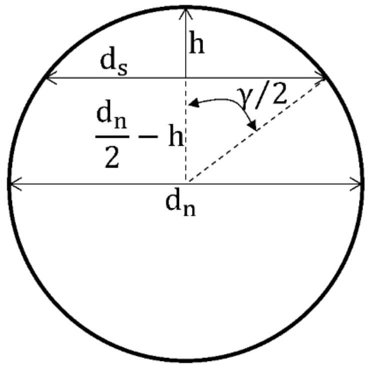

Figure S1. Representation of the TKKD circular node. The height of the cup, the strut diameter, the node diameter and the angle between the struts are indicated.

Therefore, the volume of the node is given by the volume of a sphere of diameter d_n, minus four times the volume of the spherical cap:

$$V_{node} = \frac{\pi}{6} d_n^3 - 4 \cdot \pi h^2 \left(\frac{d_n}{2} - \frac{h}{3} \right) = \left(\frac{\sqrt{2}}{3} - \frac{\sqrt{3}}{4\sqrt{2}} \right) \pi d_s^3 \quad \text{(S6)}$$

In complete analogy, the surface of the node is given by the surface of the sphere of diameter d_n, minus four times the surface of a spherical cap:

$$S_{node} = \pi d_n^2 - 4 \cdot \pi h d_n = \left(\sqrt{3} - \frac{3}{2} \right) \pi d_s^2 \quad \text{(S7)}$$

The volume of the strut is equal to the volume of a cylinder having diameter d_s and length l_s. As the struts merge in the node, the contribution of the junction must be subtracted from the volume of the cylinder. This contribution is given by two times the volume of a cylinder with diameter d_s and length equal to the thickness of the node, i.e. $\frac{d_n}{2} - h$ as shown in Figure S1. Hence, the volume of the strut can be written as follows:

$$V_{strut} = \frac{\pi}{4} d_s^2 \left(l_s - 2 \left(\frac{d_n}{2} - h \right) \right) = \frac{\pi}{4} d_s^2 \left(\frac{\sqrt{2}}{4} d_c - \frac{d_s \sqrt{2}}{2} \right) \quad \text{(S8)}$$

The surface of the strut can be derived along the same lines:

$$S_{strut} = \pi d_s \left(l_s - 2\left(\frac{d_n}{2} - h\right)\right) = \pi d_s \left(\frac{\sqrt{2}}{4} d_c - \frac{d_s\sqrt{2}}{2}\right) \tag{S9}$$

Finally, the volume of the TKKD unit cell is given by the edge length:

$$V_{cell} = 8\sqrt{2} \cdot l_s^3 = \frac{d_c^3}{2} \tag{S10}$$

Eqs. (S1), (S4), (S8) and (S10) can be combined to obtain the following expression:

$$\varepsilon = 1 - \frac{\left(\sqrt{2} - \frac{3}{2}\sqrt{6}\right)\pi d_s^3 + \frac{3}{2}\sqrt{2}\pi d_s^2 d_c}{d_c^3} \tag{S11}$$

In complete analogy, Eqs. (S2), (S7), (S9), (S10) can be combined to give:

$$S_v = \frac{\left(12\sqrt{3} - 18 - 12\sqrt{2}\right)\pi d_s^2 + 6\sqrt{2}\pi d_s d_c}{d_c^3} \tag{S12}$$

The analytical model is validated against the geometry of virtually generated structures. For this purpose, CAD models are generated by merging solid primitives, e.g., cylinders for struts and spheres for nodes, with suitable discretization to obtain a smooth surface and avoid any surface irregularity which may affect the properties evaluation. Hence, virtually generated models provide the most accurate data, comparable to the properties evaluated on real manufactured samples.

Figure S2 shows two parity plots where the geometrical properties evaluated on CAD models are compared to the predictions of the TKKD geometrical model expressed by Eqs. (S11), (S12), proving the high accuracy of the derived model in the range of void fractions of interest for engineering applications (i.e. $0.7 < \varepsilon < 0.95$). The porosities evaluated on the CAD models (Figure S2 *(a)*) superimpose the nominal void fraction line with a maximum error attaining to 0.5% for $\varepsilon = 0.7$, whereas the specific surface areas evaluated on the CAD models (Figure S2 *(b)*) are in perfect agreement with the corresponding specific surface area predictions with a maximum error of 1.2%

for ε = 0.8. compares the TKKD unit cell model proposed by Horneber[2] and the geometrical properties evaluated on CAD models, as well. Whereas the model shows an excellent agreement with the evaluated properties at sufficiently high porosity (~ε > 0.85), significant deviations in the predictions are apparent at decreasing porosity, with a maximum error attaining to 5.3% in the porosity evaluation for ε = 0.67 and 1.7% in the specific surface area evaluation for ε = 0.79.

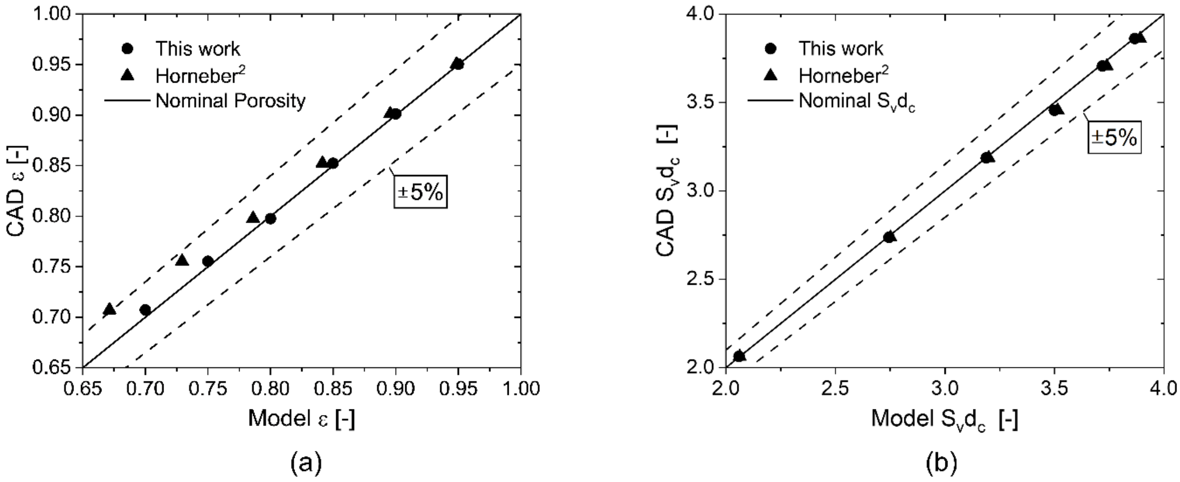

Figure S2. *(a)* Porosity evaluated on the CAD models against the nominal value. *(b)* Specific surface area evaluated on the CAD model against the nominal value.

2. Mesh generation

The definition of a proper meshing procedure is crucial to accurately describe the convective transport properties inside POCS. The computational domains are generated starting from a CAD file by means of snappyHexMesh in-built utility of the OpenFOAM framework. The CAD generation is performed by using OpenSCAD, an open-source script-based software. The snappyHexMesh utility operates starting from a uniform background mesh, refines the region of the computational domain around the CAD surface using the cut-cell approach according to the user settings and lastly snaps the mesh surface on the CAD file. Figure S3 shows a typical computational domain, made of 3 unit

cells along the streamwise direction and 1 unit cell along the transversal one. The implementation of periodic boundary conditions requires corresponding inlet and outlet sections of the domains, separated by a distance equal to an integer multiple of the cell size, and corresponding side walls, two by two coupled and separated by a distance equal to an integer multiple of the cell size as well.

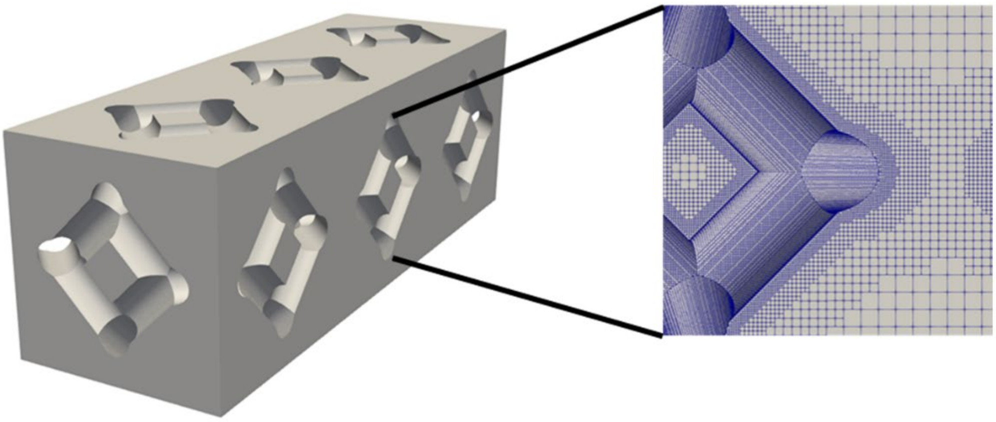

Figure S3. An example of generated computational domain for the parametric analysis of convective transport properties in POCS with a detail of the mesh discretization.

To implement cyclic boundary conditions, snappyHexMesh requires not only the domain side boundaries to be topologically matching, but also to be symmetrical planes. In this work, the TKKD and the Diamond unit cell POCS have been considered. Whereas each TKKD unit cell side boundary already constitutes a symmetrical plane (Figure S4*(a)*), this condition does not hold for the Diamond unit cell. Instead, the symmetry planes can be identified as any of the planes parallel to the diagonals of the unit cell cross section (Figure S4*(b)*). The element represented in Figure S4*(b)*, having cross section size equal to $\sqrt{2}d_c$, retains all the geometrical relevant features such as porosity and wetted surface area. Furthermore, the Diamond structure may be generated from its periodic repetition. Hence, it is considered as the minimum sized domain in the REV assessment performed in Section 4.1.

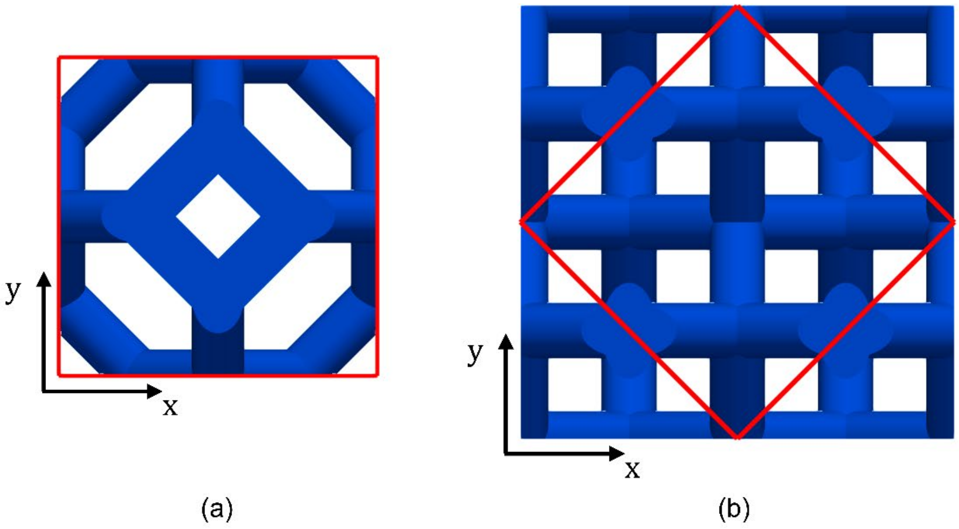

Figure S4 *(a)* TKKD and *(b)* Diamond unit cells projection on a plane orthogonal to the streamwise direction. The unit cells cross sections are marked in red.

3. Periodic boundary conditions for 3D flows

Periodic boundary conditions were first introduced by Patankar et al.[3] to model incompressible flows in ducts having streamwise periodic changing cross sections, simplified as 2D cases. The methodology is hereby extended to model reacting compressible flows in the three-dimensional space.

Periodic boundary conditions are implemented considering a computational domain constituted by an integer number of unit cells with size d_c. Letting z be the streamwise direction and x, y the transverse coordinates, the domain can be topologically described by $X_1(y,z) \leq x \leq X_2(y,z)$ and $Y_1(x,z) \leq y \leq Y_2(x,z)$, with X_1, X_2, Y_1 and Y_2 representing the coordinates of the domain side boundaries and the solid wall boundaries (see Figure S5).

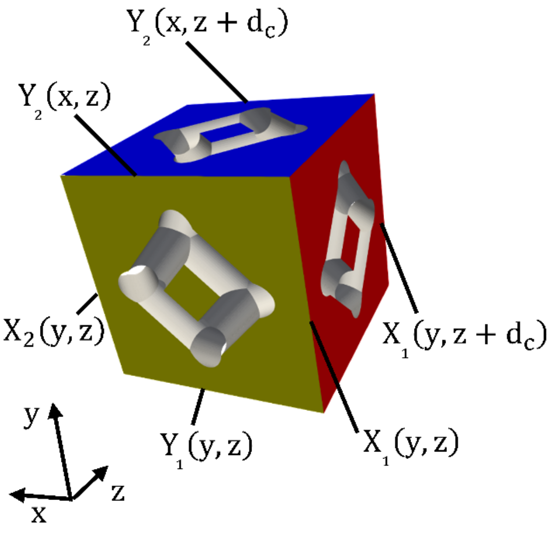

Figure S5. Reference system based on a typical computational domain constituted by a TKKD unit cell.

Consequently, for a periodic structure with periodically changing cross section, the fully developed flow field follows a periodic pattern with period d_c:

$$\rho u_x(x,y,z) = \rho u_x(x,y,z+d_c) = \rho u_x(x,y,z+2\cdot d_c) = \ \dots \\ = \ \rho u_x(x,y,z+n\cdot d_c) \tag{S13}$$

$$\rho u_y(x,y,z) = \rho u_y(x,y,z+d_c) = \rho u_y(x,y,z+2\cdot d_c) = \ \dots \\ = \ \rho u_y(x,y,z+n\cdot d_c) \tag{S14}$$

$$\rho u_z(x,y,z) = \rho u_z(x,y,z+d_c) = \rho u_z(x,y,z+2\cdot d_c) = \ \dots \\ = \ \rho u_z(x,y,z+n\cdot d_c) \tag{S15}$$

To ensure a net mass flow along the axial direction, it is necessary that the pressure field decreases along the axial direction. Therefore, the pressure field does not follow the same periodic condition expressed for the velocity field. However, the pressure in cross sections situated at a distance equal to d_c necessarily have the same shape and differ from a constant value due to the periodic profile. In this view, the pressure drop across each cell is constant as reported in Eq. (S20).

$$p(x,y,z) - p(x,y,z+d_c) = p(x,y,z+d_c) - p(x,y,z+2\cdot d_c) = \cdots$$
$$= p(x,y,z+(n-1)\cdot d_c) - p(x,y,z+n\cdot d_c) = C \quad \text{(S16)}$$

where C is the pressure drop across a unit cell. The periodic fully developed flow profile can either be simulated by imposing a driving force, that is the pressure gradient C given by Eq. (S20), which generates the motion of the fluid at a corresponding Reynolds number a priori unknown, or by imposing a fixed mass flowrate (fixed Reynolds number) and determining the pressure gradient afterwards. In our work, the second approach is adopted.

In the fully developed flow, the description of the chemical species mass concentration profiles would require the introduction of a source term to account for the reactants and products consumption and generation. However, the mass concentration profiles in cross sections situated at a distance equal to integer multiples of d_c necessarily assume the same shape and differ by a scaling factor which accounts for the reaction. Introducing the normalized mass concentration:

$$\omega_i^*(x,y,z) = \frac{\omega_i(x,y,z) - \omega_i^B(\infty)}{\omega_i^B(z) - \omega_i^B(\infty)} \; where \; \omega_i^B(z) = \frac{\int_A (\rho u \omega)|_z dA}{\int_A (\rho u)|_z dA} \quad \text{(S17)}$$

where $\omega_i^B(\infty)$ represents the species mass concentration at the thermodynamic equilibrium.

The periodicity condition of fully developed species concentration profile requires identical shapes of the normalized mass concentration profiles at successive locations separated by the distance d_c, and thus the periodicity condition may be expressed as:

$$\omega_i^*(x,y,z) = \omega_i^*(x,y,z+d_c) = \omega_i^*(x,y,z+2\cdot d_c) = \cdots = \omega_i^*(x,y,z+n\cdot d_c) \quad \text{(S18)}$$

In the analysis of the convective heat transfer, a prescribed wall temperature condition is imposed to achieve a complete analogy to the convective mass transfer case. The periodicity condition for the temperature profile in the analysis of convective heat transfer is achieved along the same lines discussed for the mass transfer.

The normalized dimensionless temperature is employed as scaling factor:

$$T^*(x,y,z) = \frac{T(x,y,z) - T_w}{T^B(z) - T_w} \ where \ T^B(z) = \frac{\int_A (\rho u c_p T)|_z dA}{\int_A (\rho u c_p)|_z dA} \tag{S19}$$

where T_w represents the prescribed wall temperature, reached at the thermodynamic equilibrium.

In the fully developed profile, the shape of the normalized dimensionless temperature profiles at successive locations separated by the distance d_c is identical. The periodicity condition can be expressed as:

$$T^*(x,y,z) = T^*(x,y,z+d_c) = T^*(x,y,z+2\cdot d_c) = \cdots = T^*(x,y,z+n\cdot d_c) \tag{S20}$$

In this work, the periodicity conditions expressed by Eq. (S17), (S18), (S19), (S22) and (S24) were implemented and exploited to set the proper inlet conditions at the inlet boundary of the computational domains. The periodicity condition on the pressure expressed by Eq. (S20) is not used, as a prescribed mass flux is imposed at the inlet boundary of the domain instead. On the contrary, the pressure drop C is subsequently determined and the assumptions are verified.

The conditions set at the domain side boundaries X_1, X_2, Y_1, Y_2 are discussed next. As no net pressure gradient exists along the transverse coordinates, the periodicity condition can be easily implemented for all the relevant fields discussed above:

$$\varphi(X_1,y,z) = \varphi(X_2,y,z), \qquad \varphi = u,p,\omega_i,T \tag{S21}$$

$$\varphi(x,Y_1,z) = \varphi(x,Y_2,z), \qquad \varphi = u,p,\omega_i,T \tag{S22}$$

Symmetrical boundary conditions represent a potential alternative to the periodicity boundary conditions expressed by Eq. (S25), (S26). Symmetrical boundary conditions require the domain side boundaries to be symmetrical planes, and in principle do not allow the flow to cross the side boundaries and to move from one unit cell to another. Whereas this condition may hold in fully laminar conditions, it may lead to an unphysical or different solution in the transition and in the

turbulent regime; hence, in this work, the periodicity boundary conditions expressed by Eq. (S25), (S26) were implemented and imposed at the domain boundaries in the performed simulations.

The boundary conditions set at the solid wall boundary are finally discussed. A no-slip boundary condition and a zero gradient boundary condition are imposed for the velocity and the pressure, respectively. In mass transfer cases, an infinitely fast reaction is imposed at the catalytic wall, to reach a null concentration of the mass transfer limited reactant at the solid surface. Instead, in heat transfer cases, the temperature is fixed at 293.15 K at the solid wall.

4. Mesh convergence analysis

In the mesh convergence analysis, the snappyHexMesh parameters have been fixed, whereas several background mesh resolutions have been tested. In this view, the snappyHexMesh parameters have been prescribed with surface level of refinement equal to 4 to achieve a high quality of refinement along the POCS surface to accurately describe the gas-to-solid transport process.

Figure S6 *(a)* shows the trend of the Sherwood number against the mesh refinement expressed as the ratio between the POCS unit cell size d_c and the mesh cell size δ. Additionally, the pressure drops are considered as a secondary relevant parameter, due to their relation to the flow dynamics inside the structures. Figure S6 *(b)* shows the trend of the pressure drops against the ratio d_c/δ. For the TKKD unit cell POCS, the grid independence is reached with a ratio of the POCS unit cell size to the mesh cell size $d_c/\delta = 20$ up to Re = 64, whereas the finer grid with $d_c/\delta = 40$ is chosen for Re = 128, this can be ascribed to the thinner boundary layer at higher velocities which requires additional cells to be fully resolved. The same extensive analysis has been performed for the Diamond unit cell POCS as well, however the result is reported for the most stringent condition at the highest Reynolds number Re = 128. In this condition, the grid independence is reached for a ratio between the cell size and the

mesh cell size $d_c/\delta = 20$. It is worth noticing that the TKKD unit cell has thinner struts than the Diamond unit cell at same porosity, and therefore the TKKD geometry requires higher superficial velocity than the Diamond geometry at same Reynolds number. This factor plays a key-relevant role in the higher computational requirement of the TKKD geometry, as a higher resolution is required to accurately describe the flow characteristics and more precisely the boundary layer. Due to the low Reynolds numbers considered, prism layers were not added close to the wall surfaces. Despite this, the mesh near wall refinement allows to reach $y^+<1$ in each flow condition to accurately describe the transport process mainly occurring in the boundary layer. The grid independence analysis has been carried out on the representative elementary volumes (REV) described in Section 4.1.

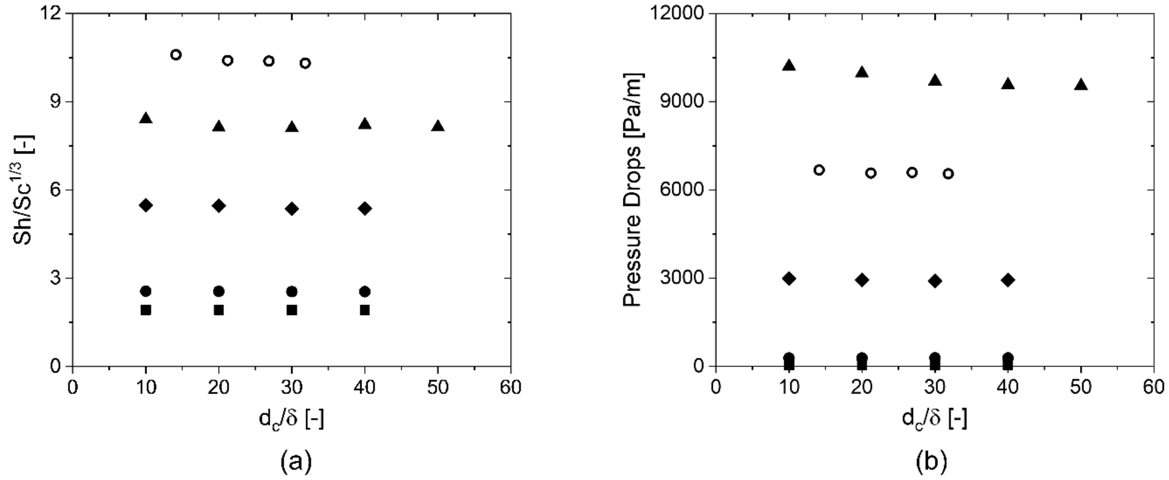

Figure S6. Mesh convergence analysis based on *(a)* the Sherwood number and *(b)* on the pressure drops for the TKKD unit cell with $\varepsilon = 0.85$ and $d_c = 3$ mm at $Re_{ds} = 4$ (squares), $Re_{ds} = 16$ (circles), $Re_{ds} = 64$ (diamonds), $Re_{ds} = 128$ (triangles); mesh convergence analysis based on *(a)* the Sherwood number and *(b)* on the pressure drops for the Diamond unit cell with $\varepsilon = 0.7$ and $d_c = 3$ mm at $Re_{ds} = 128$ (empty circles).

5. Assessment of the characteristic length (extended)

As introduced in Section 5.1, multiple choices are possible for the definition of the characteristic length, which in principle may be any geometrical parameter computed according to the equations reported in Section 3.1 - Table 1.

Lämmermann et al.[14] proposed the window diameter as characteristic length for the static liquid holdup in POCS, defined for the TKKD and the Diamond by Eqs (S27) and (S28), respectively:

$$d_w = \left(\frac{6\sqrt{3}}{\pi}\right)^{0.5} \cdot \left(\frac{\sqrt{2}}{4} d_c - \frac{\sqrt{3}}{3} d_s\right) \quad \text{(S23)}$$

$$d_w = \left(\frac{6\sqrt{3}}{\pi}\right)^{0.5} \cdot \left(\frac{\sqrt{3}}{4} d_c - \frac{\sqrt{3}}{3} d_s\right) \quad \text{(S24)}$$

The authors propose the window diameter to account for the effect of the geometrical parameters on the static liquid holdup in the two-phase pressure drop modelling, also aiming at providing a unified approach that can be applied to different POCS unit cells. In this view, the window diameter increases on increasing the cell size at fixed porosity and increasing the porosity at prescribed cell size, following the behavior of the liquid holdup. When using the window diameter as characteristic length, the transport phenomena are modelled considering the fluid as flowing in pores.

Mass transfer simulations results, reinterpreted using the window diameter as characteristic length, are reported in Figure S7. Since the window diameter is linearly dependent on the cell size and the strut diameter, which is linearly dependent on the cell size as well, the Sherwood number is only affected by the porosity, as already discussed in Section 4. The effect of the porosity is thus discussed. Figure S7 *(a)* shows the Sherwood number against the Reynolds number for the TKKD unit cell. The Sherwood number increases at increasing Reynolds numbers, consistently to what discussed in Section 4. The Sherwood number increases with the porosity, in contrast with the behavior shown in

Section 4 considering the strut diameter as characteristic length. Moreover, the Sherwood number is almost constant at $Re_{dw} > 100$, regardless of the porosity, which thus mostly influences the pure laminar regime at low Re_{dw}. Figure S7 *(b)* shows the Sherwood number against the Reynolds number for the Diamond unit cell. As for the TKKD, Sh_{dw} increases at increasing Re_{dw} and increasing porosity in all the considered flow regimes. Contrarily to the TKKD, the data do not align in the transitional laminar regime, instead, a residual dependency on the porosity is found even at high Re_{dw}.

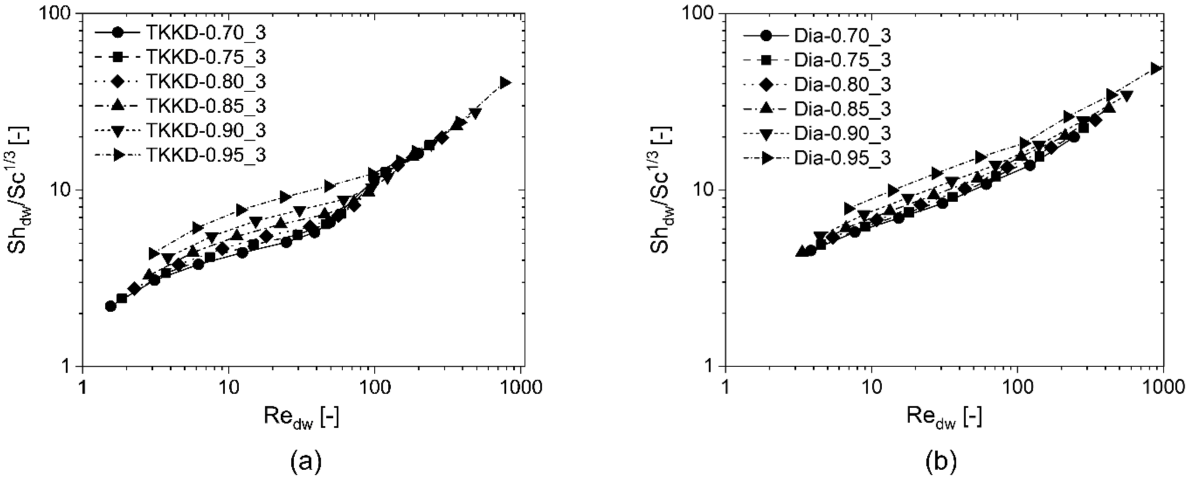

Figure S7. Sherwood numbers against the Reynolds numbers *(a)* for the TKKD unit cell and *(b)* for the Diamond unit cell using the window diameter as characteristic length. The samples are characterized by cell size d_c = 3 mm, circular struts with constant cross-section and six different porosities (ε = 0.700 circles, ε = 0.750 squares, ε = 0.800 diamonds, ε = 0.850 up-pointing triangles, ε = 0.900 down-pointing triangles, ε = 0.950 right-pointing triangles).

The non-trivial dependency of the Sherwood number on the Reynolds number and on the porosity hinder the formulation of a Sh_{dw}-Re_{dw} correlation for the two unit cells, and thus highlight the unsuitability of the window diameter as a descriptor of the gas-solid transfer properties of the examined geometries. Additionally, the choice of the window diameter contradicts the similarity

between POCS and tube banks, i.e., the nature of the fluid flowing around objects rather than flowing in pores.

Dietrich[5] proposed the hydraulic diameter for foams, defined as:

$$d_h = \frac{4\varepsilon}{S_v} \tag{S25}$$

The hydraulic diameter is generally related to the flow phenomena in ducts and honeycombs, i.e. internal flows. In this view, when considering the hydraulic diameter as characteristic length, the transport phenomena are modelled considering flow in ducts.

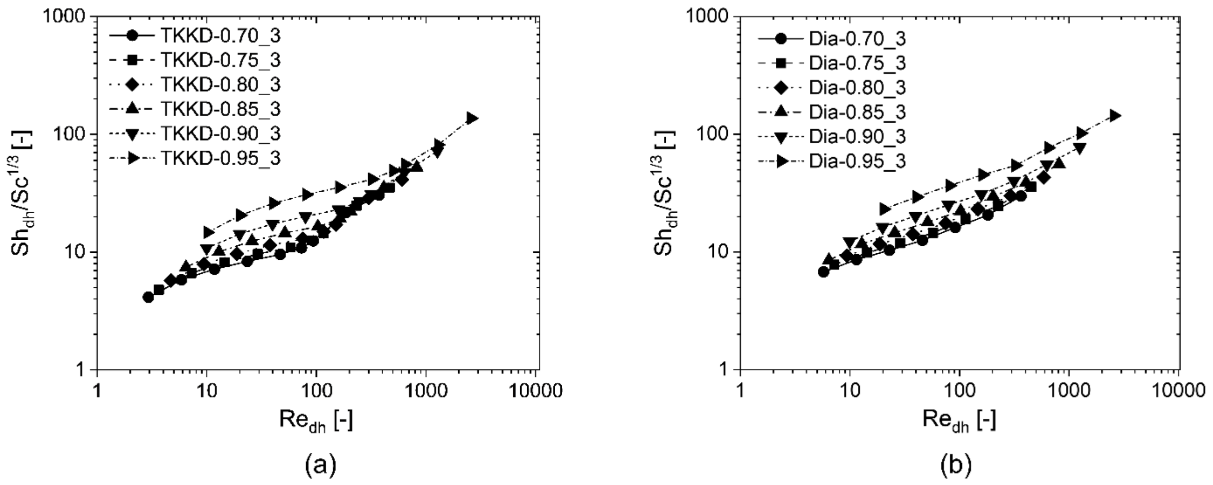

Figure S8. Sherwood numbers against the Reynolds numbers *(a)* for the TKKD unit cell and *(b)* for the Diamond unit cell using the hydraulic diameter as characteristic length. The samples are characterized by cell size d_c = 3 mm, circular struts with constant cross-section and six different porosities (ε = 0.700 circles, ε = 0.750 squares, ε = 0.800 diamonds, ε = 0.850 up-pointing triangles, e = 0.900 down-pointing triangles, e = 0.950 right-pointing triangles).

Figure S8 shows the mass transfer simulations results reinterpreted considering the hydraulic diameter as characteristic length. At given porosity, the hydraulic diameter is linearly dependent on the cell size, according to the geometrical model (see Section 3.1 - Table 1), and thus the effect of the

cell size is once again included in the Sherwood number dependency on the Reynolds number, as already discussed in Section 4 for the strut diameter. The effect of the porosity is hereby discussed. Figure S8 *(a)* shows the Sherwood number against the Reynolds number for the TKKD unit cell. The Sherwood number increases at increasing Reynolds number and increasing porosity, consistently to what previously discussed considering the window diameter as characteristic length and in contrast to what discussed in Section 4 considering the strut diameter. In analogy with the window diameter, the data appear to align at $Re_{dh} > \sim 300$ in the nonstationary laminar regime, whether a residual dependency of the Sherwood number on the porosity is shown at lower Re_{dh} in the full laminar regime.

Figure S8 *(b)* shows the Sherwood number against the Reynolds number for the Diamond unit cell. The Sherwood number increases at increasing Re_{dh} and increasing porosity. In contrast to the TKKD, the residual dependence of the Sh_{dh} on the porosity is evident in all the flow conditions. Moreover, the data appear to collapse on a single line at decreasing porosity.

Because of the non-trivial dependency of the Sherwood number on the porosity and on the Reynolds number, the hydraulic diameter is shown to be a poor descriptor of the gas-solid transfer properties of the examined geometries and is thus discarded.

Reichelt et al.[54] proposed the equivalent sphere diameter, or Sauter diameter for mass transfer in generalized porous media :

$$d_{Sauter} = \frac{6(1-\varepsilon)}{S_v} \tag{S26}$$

The equivalent sphere diameter is inherited by the description of transport phenomena in packed beds, for which a sphere-like diameter is generally adopted to formulate heat and mass transfer correlations. The porosity and the specific surface area of POCS are required to define the Sauter diameter. In this work, the porosity and the cell size are assigned, accordingly, the specific surface area is provided by the geometrical model. In this view, by combining the expressions reported in

Section 3.1 – Table 1, the Sauter diameter can be defined for the TKKD and the Diamond according to Eq. (S31) and Eq. (S32), respectively:

$$d_{Sauter} = d_s \cdot \left(\frac{3\sqrt{2}\frac{d_c}{d_s} + 2\sqrt{2} - 3\sqrt{6}}{2\sqrt{2}\frac{d_c}{d_s} + 4\sqrt{3} - 6 - 4\sqrt{2}} \right) = d_s \cdot \psi(\varepsilon) \tag{S27}$$

$$d_{Sauter} = d_s \cdot \left(\frac{3\sqrt{3}\frac{d_c}{d_s} + 2\sqrt{2} - 3\sqrt{6}}{2\sqrt{3}\frac{d_c}{d_s} + 4\sqrt{3} - 6 - 4\sqrt{2}} \right) = d_s \cdot \beta(\varepsilon) \tag{S28}$$

In Eq. (S31) and Eq. (S32), the expressions $\psi(\varepsilon)$ and $\beta(\varepsilon)$ are inherited from the geometrical models and are non-linear functions of the porosity only, being the strut diameter linearly dependent on the cell size at prescribed porosity. Noteworthy, $\psi(\varepsilon)$ and $\beta(\varepsilon)$ are weak functions of the porosity, varying between 1.5 and 2 for the two considered geometries in the considered porosity range $\varepsilon = 0.7$-0.95, accordingly, a quasi-linear relationship exists between the Sauter diameter and the strut diameter, as already observed for open-cell foams[9].

It is thus possible to revisit the heat and mass transfer correlations proposed in Section 5.2 considering the Sauter diameter as characteristic length, with different coefficients accounting for the factors $\psi(\varepsilon)$ and $\beta(\varepsilon)$. In the case of the TKKD unit cell, the functional form provided by Section 5.2, Eq. 8 is kept in the formulation of the heat and mass transfer correlation:

$$\mathrm{Sh}_{\mathrm{d_{Sauter}}}/\mathrm{Sc}^{1/3} \text{ or } \mathrm{Nu}_{\mathrm{d_{Sauter}}}/\mathrm{Pr}^{1/3} = \mathrm{A}' \cdot \mathrm{Re}^{\mathrm{m}}_{\mathrm{d_{Sauter}}} \cdot \varepsilon^{-1.5} \tag{S29}$$

The Sherwood number functional dependence on the porosity and on the Reynolds number is preserved, whereas the coefficients A' account for the linear relationship between the strut diameter and the Sauter diameter.

Along the same lines, the heat and mass transfer correlation proposed for the Diamond unit cell assumes an analogous functional form to Section 5.2, Eq. 9:

$$\mathrm{Sh}_{\mathrm{d_{Sauter}}} = \varepsilon^{-1.5} \cdot \left(\mathrm{B'} \cdot \mathrm{Re}_{\mathrm{d_{Sauter}}}^{1/3} + \mathrm{C'} \cdot \mathrm{Re}_{\mathrm{d_{Sauter}}}^{0.8}\right) \cdot \mathrm{Sc}^{1/3}$$

$$\mathrm{Nu}_{\mathrm{d_{Sauter}}} = \varepsilon^{-1.5} \cdot \left(\mathrm{B'} \cdot \mathrm{Re}_{\mathrm{d_{Sauter}}}^{1/3} + \mathrm{C'} \cdot \mathrm{Re}_{\mathrm{d_{Sauter}}}^{0.8}\right) \cdot \mathrm{Pr}^{1/3} \quad \text{(S30)}$$

The applicability ranges of the derived correlations are $1 \leq Re_{dSauter} \leq 200$, $0.7 \leq \varepsilon \leq 0.95$ and $1 \leq d_c \leq 8$ mm.

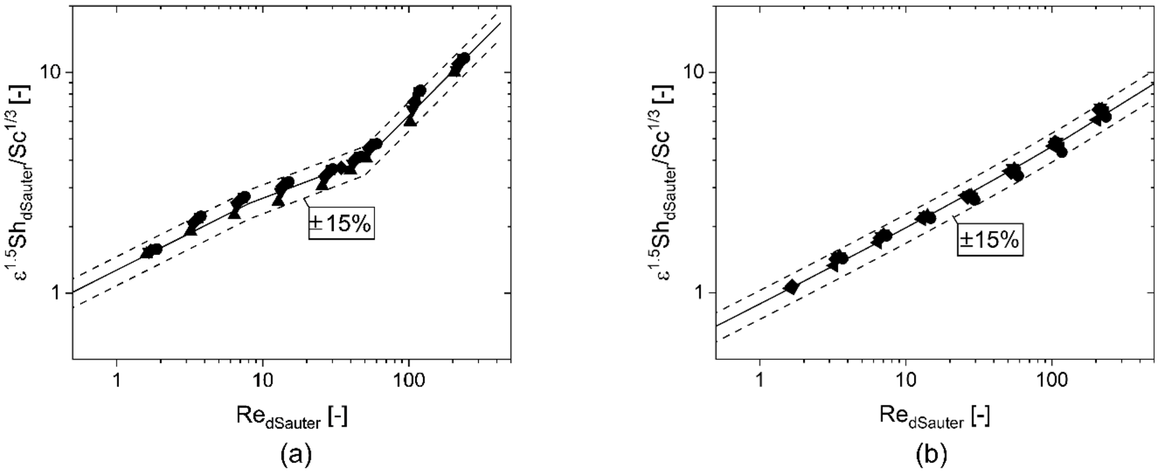

Figure S9. Mass transfer correlation *(a)* for the TKKD unit cell and *(b)* for the Diamond unit cell using the Sauter diameter as a characteristic length.

Figure S9 shows the simulation data along with the rephrased correlations *(a)* for the TKKD unit cell and *(b)* for the Diamond unit cell given by Eq. (S33) and (S34). A good agreement is apparent, with deviations below ±15% between the correlation and the data, and also proving the suitability of the Sauter diameter and its equivalence to the strut diameter in the description of the gas-solid interphase heat and mass transfer in POCS.

References

(1) Ambrosetti, M.; Bracconi, M.; Groppi, G.; Tronconi, E. Analytical Geometrical Model of Open Cell Foams with Detailed Description of Strut-Node Intersection. *Chemie-Ingenieur-Technik* **2017**, *89* (7), 915–925.

(2) Horneber, T. Thermo-Fluid Dynamic Characterization and Technical Optimization of Structured Open-Cell Metal Foams by Means of Numerical Simulation. **2015**.

(3) Patankar, S. V.; Liu, C. H.; Sparrow, E. M. Fully Developed Flow and Heat Transfer in Ducts Having Streamwise-Periodic Variations of Cross-Sectional Area. *J. Heat Transfer* **1977**, *99* (2), 180–186.

(4) Lämmermann, M.; Horak, G.; Schwieger, W.; Freund, H. Periodic Open Cellular Structures (POCS) for Intensification of Multiphase Reactors: Liquid Holdup and Two-Phase Pressure Drop. *Chem. Eng. Process. - Process Intensif.* **2018**, *126*, 178–189.

(5) Dietrich, B. Pressure Drop Correlation for Ceramic and Metal Sponges. *Chem. Eng. Sci.* **2012**, *74*, 192–199.
